# Size-selective molecular recognition based on a confined DNA molecular sieve using cavity-tunable framework nucleic acids

**DOI:** 10.1038/s41467-020-15297-7

**Published:** 2020-03-23

**Authors:** Xiaoyi Fu, Guoliang Ke, Fangqi Peng, Xue Hu, Jiaqi Li, Yuyan Shi, Gezhi Kong, Xiao-Bing Zhang, Weihong Tan

**Affiliations:** 1 Molecular Science and Biomedicine Laboratory (MBL), State Key Laboratory of Chemo/Biosensing and Chemometrics, College of Chemistry and Chemical Engineering, Hunan University, 410082 Changsha, China; 2 Institute of Cancer and Basic Medicine (IBMC), Chinese Academy of Sciences, The Cancer Hospital of the University of Chinese Academy of Sciences, 310022 Hangzhou, Zhejiang China; 3 Institute of Molecular Medicine (IMM), Renji Hospital, School of Medicine and College of Chemistry and Chemical Engineering, Shanghai Jiao Tong University, 200240 Shanghai, China

**Keywords:** DNA probes, Bioanalytical chemistry, Sensors

## Abstract

Size selectivity is an important mechanism for molecular recognition based on the size difference between targets and non-targets. However, rational design of an artificial size-selective molecular recognition system for biological targets in living cells remains challenging. Herein, we construct a DNA molecular sieve for size-selective molecular recognition to improve the biosensing selectivity in living cells. The system consists of functional nucleic acid probes (e.g., DNAzymes, aptamers and molecular beacons) encapsulated into the inner cavity of framework nucleic acid. Thus, small target molecules are able to enter the cavity for efficient molecular recognition, while large molecules are prohibited. The system not only effectively protect probes from nuclease degradation and nonspecific proteins binding, but also successfully realize size-selective discrimination between mature microRNA and precursor microRNA in living cells. Therefore, the DNA molecular sieve provides a simple, general, efficient and controllable approach for size-selective molecular recognition in biomedical studies and clinical diagnoses.

## Abstract (web summary)

Size-selective discrimination is an issue in biosensing. Here, the authors report on a size selective DNA nanocage which excludes agents based on size and protects the probes against degradation, and demonstrate the discrimination between mature and precursor miRNA.

## Introduction

Biosensing based on molecular recognition tools, such as nucleic acids and proteins, plays a critical role in biomedical studies and clinical diagnoses^1–3^. Particularly, the detection selectivity is a highly important parameter for a biosensor. The detection selectivity of current biosensors generally are based on several principles^4–7^, including base-paring principle (e.g. nucleic acids), affinity interaction (e.g. antibodies, aptamers), and so on. Though these methods have made some progress, they failed the selective and accurate biosensing for some biological targets in a specific situation where the properties of targets and analogues (such as nucleic acids sequences) are too similar to be distinguished. For example, although a number of intracellular microRNAs (miRNAs) biosensors have been developed based on sequence complementarity of miRNAs and nucleic acid probes^8–14^, their detection accuracy for mature miRNAs is affected by the presence of its precursor (precursor microRNAs, abbreviated as pre-miRNAs) since the sequence of mature miRNAs is also present in the precursors. Alternatively, considering the different length of mature miRNAs (19–23 nt) and pre-miRNAs (60–70 nt)^15^, a size-selective molecular strategy based on the size difference between targets and non-targets is promising. Unfortunately, the rational design of an artificial size-selective molecular recognition system for biological targets in living cells remains challenging, because of the difficulty in the precise and site-specific functionalization of molecular recognition elements in a precise framework with nanometer precision.

**Fig. 1:**
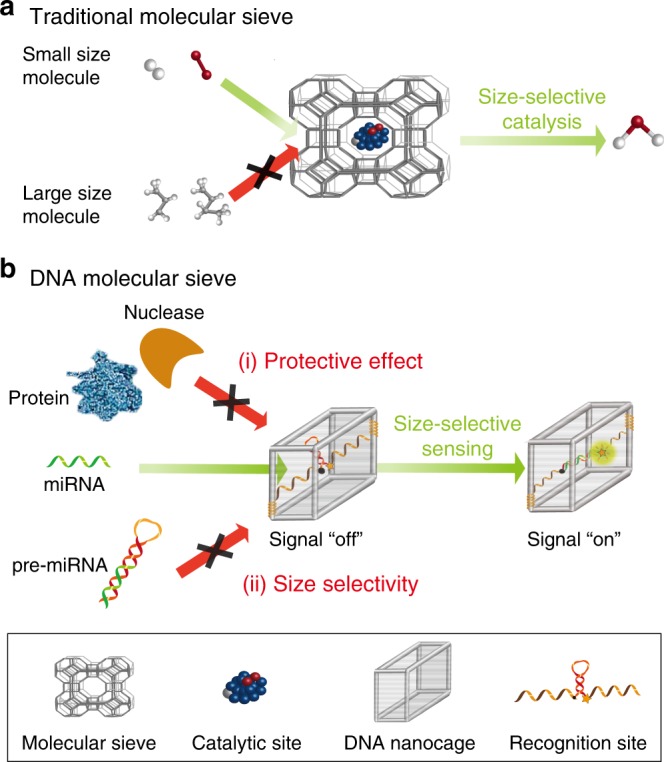
Scheme illustration of DNA molecular sieve. **a** Size-selective catalysis based on a traditional molecular sieve. **b** Size-selective molecular recognition in the confined space of DNA molecular sieve using cavity-tunable DNA nanocage framework nucleic acids. Large molecules, such as nucleases, proteins and larger analogues, are prohibited from contacting with inner recognition sites, while small target molecules are able to enter the cavity for efficient molecular recognition.

In catalysis science, an important example for size selectivity is the size-selective catalysis (or shape-selective catalysis) in the pores of molecular sieve, which is natural or synthetic zeolite materials engineered with precise and uniform pores^16,17^. That is, in a molecular sieve-based catalyst, active catalytic sites are encapsulated into the inner cavity of molecular sieves with diameters that are similar to those of targeted small-molecule substrates. As such, larger molecules are prohibited from contacting with inner active catalytic sites, while smaller target molecules are able to enter the pores to participate in chemical reaction (Fig. 1a). Benefiting from the size-selective molecular recognition in the confined space of molecular sieves, size-selective catalysis is able to improve catalysis selectivity and efficiency, as well as protect the catalyst sites from catalyst poisoning, thus leading to the wide application of molecular sieve-based catalyst in industrial catalysis^18–22^.

Inspired by this interesting and important approach, herein we developed a DNA molecular sieve for the size-selective recognition to improve the biosensing selectivity and accuracy. Framework nucleic acids, which involve the rational design of DNA nanostructures based on the high predictability of Watson-Crick base pairing, provide the ability for high controllability and nanometer precision^23–31^. In particular, with the programmable and precise control of their size, shape, and binding sites, framework nucleic acids enable the precise and site-specific functionalization of guest objects^32–41^, including inorganic nanoparticles, nucleic acids, enzymes, hydrophobic micelles, and so on. Besides, the framework nucleic acids could also protect their cargoes^42–44^. However, the biosensing application of framework nucleic acids based on size-selective recognition has not been reported. Functional nucleic acids (FNAs)^4,45^, such as aptamers, DNAzymes, and molecular beacons, provide excellent molecular recognition ability, and play important roles in biological analysis^46–50^. Combining the advantage of these two kinds of nucleic acids, we herein develop a DNA molecular sieve for size-selective recognition through the site-specific encapsulation of functional nucleic acids in cavity-tunable framework nucleic acids in an efficient and controlled manner. Based on this strategy, the DNA molecular sieve not only can protect DNAzyme from nuclease digestion and non-specific protein absorption, but also exhibit the versatility for other functional nucleic acids (eg. aptamer and molecular beacon). Most important, the DNA molecular sieve can achieve size-selectivity biosensing, which is capable of distinguishing mature microRNA from precursor microRNA.

As shown in Fig. 1b, the DNA molecular sieve is constructed using a strategy based on the addressable integration of active molecular recognition sites in the cavity of DNA nanocage framework for inner encapsulation design (CONFINED strategy). The CONFINED strategy is applied to develop a series of functional nucleic acid probes (CONFINED-FNAs) including DNAzymes, aptamers and molecular beacons for molecular recognition of a wide range of biological targets. With the CONFINED strategy, size dependent target accessibility of the nanocage is achieved. That is, large molecules, such as nucleases, proteins and larger analogues, are prohibited from making contact with inner FNAs, while small target molecules are able to enter the cavity for efficient molecular recognition. As a result, CONFINED-FNAs can protect FNAs from endogenous nuclease digestion and nonspecific proteins binding, ensuring their stable and accurate biosensing in living cells. Furthermore, since the cavity of framework nucleic acids can be precisely tuned to distinguish target molecules from their larger analogues, CONFINED-FNAs provides the desirable capability in size-selective sensing of specific biological targets. For example, the size-selective discrimination of intracellular mature microRNA from pre-microRNA with longer sequence is demonstrated here. With these features, the DNA molecular sieve is able to achieve the size-selective and accurate sensing of FNAs probes in a precise manner. Meanwhile, it provides a blueprint for size-selective molecular recognition in biomedical studies and clinical diagnoses.

## Results

### Design of cavity-tunable framework nucleic acids

**Fig. 2:**
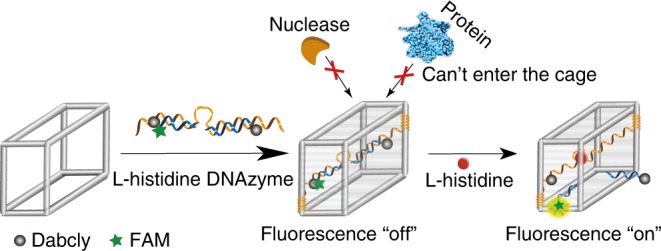
Scheme illustration of CONFINED-DNAzyme. The design of DNAzyme encapsulated in a cavity-tunable framework nucleic acid.

DNAzymes represent one common kind of functional nucleic acid that was first employed as a model to demonstrate the concept of size-selective recognition based on a cavity-tunable framework nucleic acids. As illustrated in Fig. 2, in the CONFINED-based DNAzyme probe (CONFINED-DNAzyme), DNAzyme (l-histidine DNAzyme^51^ in this case) with a quencher (DABCYL) was assembled into the inner cavity of DNA nanocages, which could quench the fluorophore (FAM) labeled on the substrate strand. In the presence of small-molecule target (histidine in this case), the DNAzyme cleaves the substrate strand to separate the quencher and fluorophore, thus triggering the recovery of fluorescence signal. Importantly, owing to the size-selective permeability of the DNA nanocage shell, the CONFINED-DNAzyme can avoid contact with macromolecules, such as nucleases and proteins, thus protecting the DNAzyme from rapid nuclease degradation and nonspecific protein binding.

### Preparation and characterization of framework nucleic acids

**Fig. 3:**
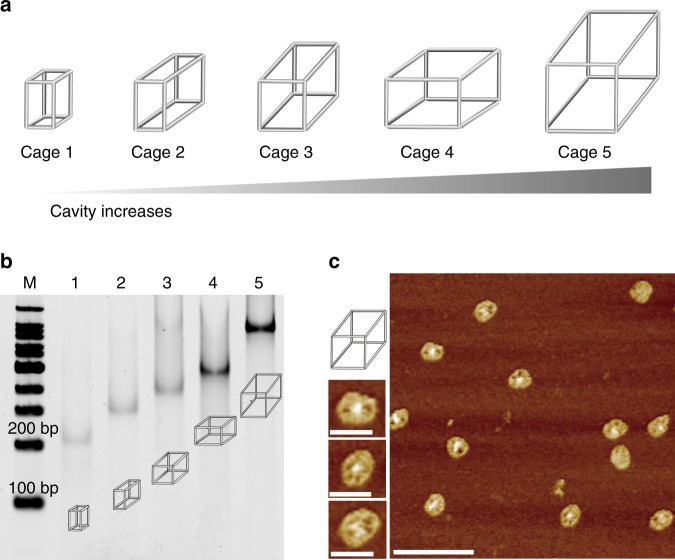
Design and characterization of DNA nanocages with different cavity sizes. **a** Schematic illustration of DNA nanocages construction. The cavity of DNA nanocages gradually increases from cage 1 to cage 5. **b** N-PAGE characterization of DNA nanocages from cage 1 (lane 1) to cage 5 (lane 5). **c** Atomic force microscopy characterization of DNA nanocages (Cage 5). The scale bars are 100 and 30 nm in large and small imaging, respectively. Source data are provided as a Source Data file.

To investigate the cavity-tunable effect, we designed and prepared five different sizes of framework nucleic acids (DNA nanocages here, shown in Fig. 3a)^41^. Based on the DNA’s helical twist of 10.5 bases per turn, the theoretical size ranking of these DNA nanocages is as follows: Cage 1 (3.4 nm × 7.1 nm × 3.4 nm) < Cage 2 (3.4 nm × 7.1 nm × 7.1 nm) < Cage 3 (7.1 nm × 7.1 nm × 7.1 nm) < Cage 4 (10.5 nm × 7.1 nm × 7.1 nm) < Cage 5 (10.5 nm × 10.5 nm × 10.5 nm). We first verified if each kind of DNA nanocage could be successfully assembled with the stepwise addition of each DNA strand (100 nM) by native polyacrylamide gel electrophoresis (N-PAGE, Supplementary Figs. 1–5). Moreover, with the gradual increase of the cavity size from Cage 1 to Cage 5, a gradual reduction of electrophoretic mobility of DNA nanocages was observed (Fig. 3b), relative to the increased molecular mass of DNA nanostructures. Dynamic light scattering (DLS) also confirmed the gradual increase of hydration radius for DNA nanocages (Supplementary Fig. 6). In addition, atomic force microscopy (AFM) was used to characterize the structure of the cage (e.g. Cage 5). The nanostructures with cavity were clearly observed in the AFM imaging (Fig. 3c), verifying the successful formation of DNA nanocages with cavities.

### Protection effect of framework nucleic acids

**Fig. 4:**
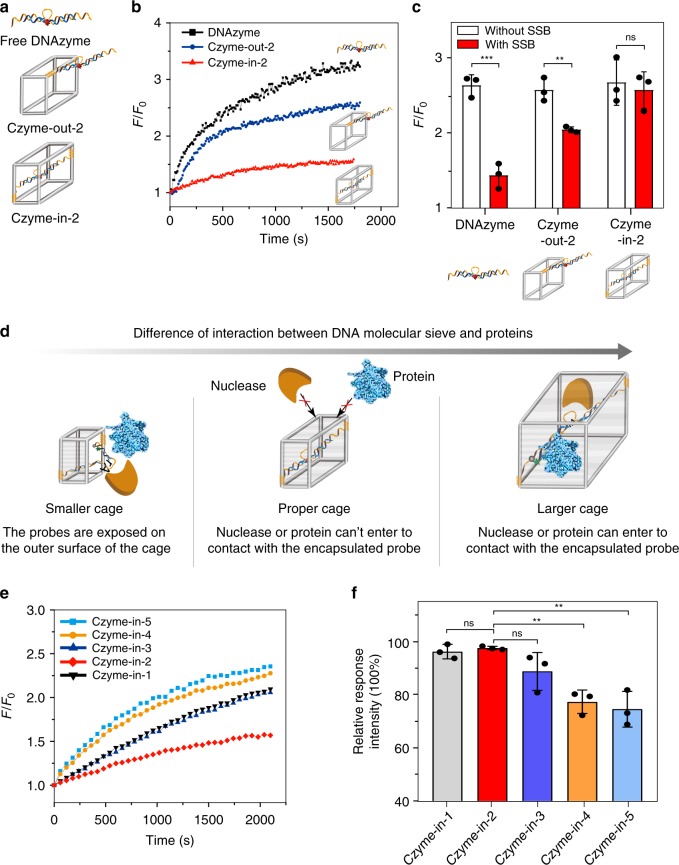
The size-dependence protective performance of DNAzyme encapsulated in the DNA nanocages. **a** Schematic illustration of three kinds of DNAzyme probes: free DNAzyme, DNAzyme connected to the outside frame of Cage 2 (Czyme-out-2), and DNAzyme encapsulated inside Cage 2 (Czyme-in-2). **b** Time-dependent fluorescence changes of different probes after the addition of nuclease (F_0_ fluorescence intensity without DNase I, F fluorescence intensity with DNase I). **c** Fluorescence response of free DNAzyme, Czyme-out-2 and Czyme-in-2 to l-histidine treated with and without SSB (F_0_ fluorescence intensity without l-histidine, F fluorescence intensity with l-histidine); Data are presented as mean values ± s.d. (*n* = 3); ***p = 0.00070 < 0.001, **p = 0.0046 < 0.01, ns = 0.6800 > 0.05 (not significant), by two-tailed unpaired Student’s *t*-test. **d** Schematic illustration of the cavity effect; **e** Time-dependent fluorescence changes of different cage sizes after the addition of nuclease (F_0_ fluorescence intensity without DNase I, F fluorescence intensity with DNase I). **f** Relative response intensity of different cage sizes to l-histidine after treatment with single-strand binding protein (SSB) (“Relative response intensity” refer to the ratio of (F/F_0_) with and without SSB; F: fluorescence intensity with l-histidine, F_0_ fluorescence intensity without l-histidine); Data are presented as mean values ± s.d. (*n* = 3); **p = 0.0014 (0.0040) for Czyme-in-4 (Czyme-in-5) < 0.01, ns = 0.43 (0.10) for Czyme-in-1 (Czyme-in-3) > 0.05 (not significant), by two-tailed unpaired Student’s *t*-test. Source data are provided as a Source Data file.

Generally, the intracellular application of FNAs, such as DNAzymes, suffers from nuclease degradation and nonspecific protein binding in living cells, which is one of the bottleneck of current nucleic acids probes^52^. To demonstrate the feasibility of using DNA molecular sieve to solve this challenging problem, we first investigated the protective effect of framework nucleic acids on the DNAzyme. As shown in Fig. 4a, the model system included three forms of DNAzyme: free DNAzyme, DNAzyme attached to the outside frame of DNA Cage 2 (Czyme-out-2, Supplementary Fig. 7), and DNAzyme encapsulated in the cavity of DNA Cage 2 (Czyme-in-2, Supplementary Fig. 2). Before investigating the protective effect of framework nucleic acids, we firstly demonstrated that the DNA nanocages are more stable than double-stranded strands in the presence of nuclease (e.g., DNase I^53^) (Supplementary Fig. 8). A binding test confirmed the addressable modification of DNAzyme inside and outside the cavity of DNA nanocage in Czyme-in-2 and Czyme-out-2, respectively (Supplementary Fig. 9). The sensing performance of these three forms of DNAzyme (final concentration is 10 nM and molar ratio of probe to nanocage is 1:1) was evaluated in the presence of nuclease (e.g., DNase I) or proteins (e.g., single-strand binding protein, SSB^54^). The DLS result (Supplementary Fig. 10) indicated the size of DNase I and SSB to be 6.5 ± 2.0 and 7.5 ± 2.0 nm, respectively, which is in agreement with their three-dimensional dimensions^55–57^. Thus, DNA Cage 2 was chosen based on its suitable size. As shown in Fig. 4b, in the presence of 1 U mL^−1^ DNase I, the fluorescence intensity of free DNAzyme significantly increased, indicating its poor stability. Although Czyme-out-2 exhibited better stability than free DNAzyme, it was still gradually digested. In contrast, Czyme-in-2 showed the smallest fluorescence change, suggesting the significantly improved stability of Czyme-in-2 in the presence of nuclease. We also investigated the response ability of Czyme-in-2 after treated with DNase I for longer time points by fluorescence spectra (Supplementary Fig. 11) and native-PAGE analysis (Supplementary Fig. 12). The result proved that Czyme-in-2 was resistant to enzyme degradation and still retained response activity. Furthermore, we compared the nonspecific protein binding property using SSB. As shown in Fig. 4c and the fluorescence spectra in Supplementary Fig. 13, the responsive signal to l-histidine of free DNAzyme and Czyme-out-2 obviously decreased in the presence of SSB. This could be explained by the nonspecific binding of SSB protein to the single-strand part of DNAzyme, thus inducing the increase of background signal. In contrast, the target response ability of Czyme-in-2 showed little change in the presence of SSB, indicating that Czyme-in-2 could protect DNAzyme from nonspecific binding of proteins. Subsequently, further stability testing in cell lysate, a more realistic environment, also confirmed the desirable resistance property of Czyme-in-2 (Supplementary Fig. 14). These results convincingly proved that the CONFINED strategy was able to effectively improve biostability and reduce nonspecific protein binding of DNAzyme, thus avoiding false results in complex biological systems.

### Size dependence of CONFINED-based DNAzyme

Since the sizes of nucleases and proteins are generally larger than those of small-molecule targets, the mechanism of improved biostability and reduced nonspecific protein binding of Czyme-in could be mainly attributed to the size-selective permeability of the DNA nanocages. As shown in Fig. 4d, the cavity of an optimal DNA nanocages should be smaller than the hydrodynamic radius of nucleases or proteins, but is still large enough to fully accommodate the DNAzymes. Thus, the DNA nanocages could successfully intercept nucleases and proteins, while permit small-molecule targets to make contact with the DNAzymes (middle column). However, if the cavity is too large, then the nucleases and proteins would have free access to the cages, allowing them to affect the performance of the encapsulated DNAzyme (right column). If the cavity of the nanocages is too small to completely encapsulate the DNAzymes, then exposed FNAs would obviously be affected by nuclease digestion and nonspecific protein binding outside the nanocages (left column). In order to confirm this set of presumptions, the anti-nuclease and anti-protein nonspecific binding abilities of DNAzyme encapsulated in nanocages (final concentration is 10 nM and molar ratio of probe to nanocage is 1:1) of different sizes were investigated. Anti-nuclease ability was tested by incubating the same concentration of DNase I (1 U mL^−1^) with different cages. Anti-protein nonspecific binding was estimated by the relative response intensity of probes to l-histidine with and without SSB. As shown in Fig. 4e, f, DNAzyme in Czyme-in-2 exhibited the best anti-nuclease and anti-protein nonspecific binding abilities. However, the performance of DNAzyme in smaller cages (Czyme-in-1) and larger cages (Czyme-in-3 to Czyme-in-5) were not as stable as Czyme-in-2 in the presence of DNase I and SSB. These results suggest that Czyme-in-2, whose cavity is most comparable to the hydrodynamic radius of proteins, is the most suitable nanocage for encapsulation of DNAzyme. Therefore, these results confirmed that the size-selective permeability of DNA nanocages could provide improved biostability and reduced nonspecific protein binding of FNAs.

### The biosensing application of CONFINED-based DNAzyme

**Fig. 5:**
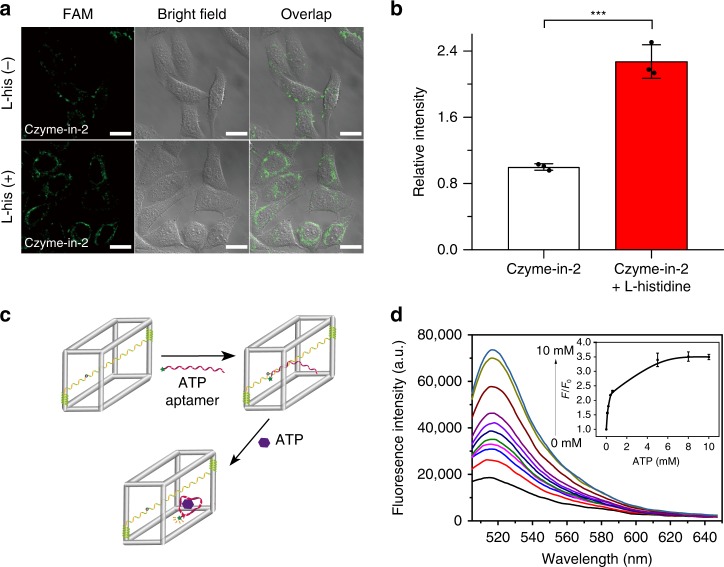
The biosensing application of CONFINED-based biosensor. **a** Fluorescence confocal imaging of HeLa cells incubated with Czyme-in-2 without and with l-histidine, respectively. The scale bars are 20 μm. **b** The relative fluorescence intensity of Czyme-in-2 confocal imaging that quantified by ImageJ; Data are presented as mean values ± s.d. (*n* = 3), Student’s *t* test, ***p = 0.00043 < 0.001, by two-tailed unpaired Student’s *t*-test. **c** Schematic illustration of ATP aptamer encapsulated in the DNA cages (Cage-apt-in-2). **d** Fluorescence response in the presence of different concentrations of ATP, ranging from 0 to 10 mM. Inset: relationship between fluorescence enhancement and concentrations, data are presented as mean values ± s.d. (*n* = 3). Source data are provided as a Source Data file.

Taking advantages of the improved biostability and reduced nonspecific protein binding, Czyme-in-2 is expected to realize intracellular biosensing of targets with better stability and accuracy. We first evaluated the sensing performance of Czyme-in-2 in buffer. First, the response kinetics of Czyme-in-2 was proved to be similar to that of free l-histidine-specific DNAzyme probes (Supplementary Fig. 15), indicating that the DNAzyme encapsulated in the cage retains similar catalytic activity. Supplementary Fig. 16a shows the fluorescence response of Czyme-in-2 to different concentrations of l-histidine (0–10 mM), confirming the sensing ability of Czyme-in-2, which further revealed excellent selective detection of l-histidine compared with other amino acids (Supplementary Fig. 16b). These results clearly demonstrated that Czyme-in-2 maintains its catalytic function for the sensitive and selective detection of its target. We next investigated the intracellular biosensing performance of Czyme-in-2 compared with the free DNAzyme transfected by liposome in HeLa cells. The free DNAzyme showed negligible fluorescence enhancement in the presence of l-histidine owing to the high background signal of probes (Supplementary Fig. 17), indicating its poor stability and unsatisfactory sensing ability in the complex intracellular environment. On the contrary, Czyme-in-2 showed significant signal enhancement in the presence of l-histidine compared to negative cells without l-histidine treatment (Fig. 5a, b). The *z*-axis scanning imaging further confirmed that the Czyme-in-2 could response inside the cells (Supplementary Fig. 18). These results suggested that DNAzyme encapsulated in the DNA nanocages could provide more stable and accurate biosensing in complex biological systems. Furthermore, the structural integrity and nanoscale size of DNA nanocages provided Czyme-in-2 the ability of cellular uptake (Supplementary Fig. 19), thus avoiding the time-consuming and complex transfection process. In addition, a standard MTS assay showed that the DNA nanocages exhibited excellent biocompatibility (Supplementary Fig. 20).

### Universality of CONFINED strategy

Based on its facile design, our CONFINED strategy could serve as a general approach for the development of a variety of FNA biosensors. For example, in the CONFINED-based aptamer, aptamer probes encapsulated in the DNA nanocages (termed as Cage-apt-in, Fig. 5c and Supplementary Fig. 21) were also designed. The ATP-binding aptamer^58^ was chosen as model. Since the probe encapsulated in Cage 2 (termed Cage-apt-in-2) showed better biosensing performance (Supplementary Fig. 22), we encapsulated the ATP aptamer in Cage 2 for further experiments. As expected, the Cage-apt-in-2 enabled the sensitive and selective sensing of ATP in vitro (Fig. 5d and Supplementary Fig. 23). The liner range of Cage-apt-in-2 is from 0.1 to 10 mM in the form of common logarithm (Supplementary Fig. 24). The native-PAGE analysis also demonstrated the Cage-apt-in-2 kept the responsive ability to ATP in the presence of nuclease (Supplementary Fig. 25). Moreover, our method is compared to a standard assay method based on UV-vis absorption spectrometry^59^ (Supplementary Fig. 26), indicating its accuracy detection ability for ATP assay (Supplementary Table 1).Besides, the Cage-apt-in-2 could also achieve the biosensing of endogenous ATP changes during drug treatment of cells (Supplementary Fig. 27). Therefore, the successful encapsulation of ATP aptamer probes suggested that this CONFINED strategy could be a versatile method for developing intracellular FNA biosensors.

### Size-selective sensing of mature miRNAs

**Fig. 6:**
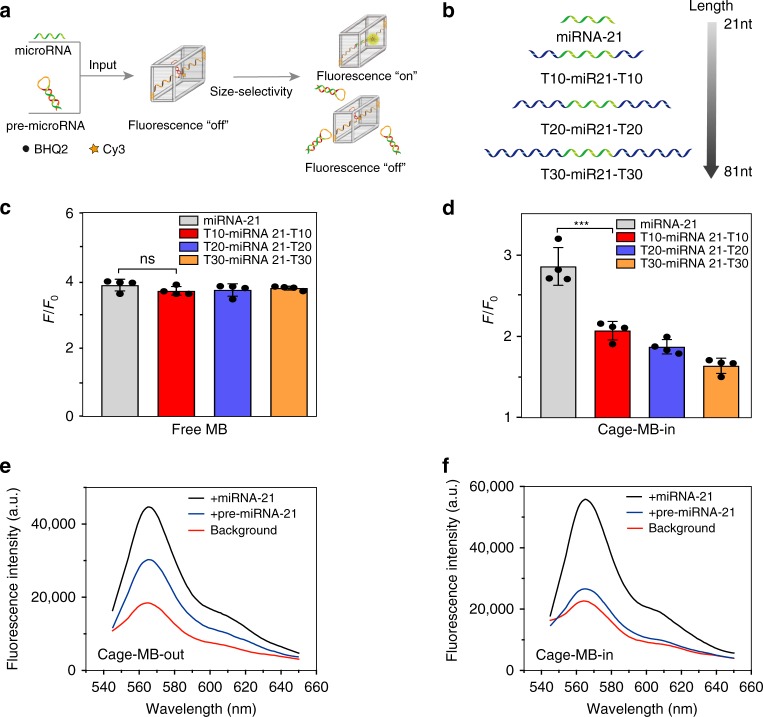
Size-selective sensing of mature miRNAs. **a** Schematic illustration of size-selective molecular recognition to distinguish mature microRNA from precursor microRNA. **b** Schematic illustration of different lengths of nucleic acid targets containing the same recognition sequence of *miRNA-21*. **c**, **d** Fluorescence response of free MB (**c**) and Cage-MB-in (**d**) to the nucleic acid targets with different lengths (F: fluorescence intensity in the presence of target, F_0_: fluorescence intensity in absence of target) **;** Data are presented as mean values ± s.d. (*n* = 4), ns = 0.17 (0.30, 0.37) for T10-miRNA 21- T10 (T20-miRNA 21- T20, T30-miRNA 21- T30) to Free MB > 0.05 (not significant), ***p = 0.00089 (0.00021, 0.000068) for T10-miRNA 21-T10 (T20-miRNA 21-T10, T30-miRNA 21-T30) to Cage-MB-in <0.001, by two-tailed unpaired Student’s *t*-test. **e**–**f** Fluorescence study of the fluorescence response of Cage-MB-out (**e**) and Cage-MB-in (**f**) to *pre-miRNA-21* and mature *miRNA-21*. Source data are provided as a Source Data file.

Since the cavities of framework nucleic acids can be precisely tuned to distinguish target molecules from their analogues, the DNA molecular sieve provides the desirable ability of size-selective sensing of biological targets with specific sizes. As proof of concept, the size-selective sensing of microRNA and its precursor was demonstrated using CONFINED strategy-based molecular beacon (CONFINED-MB). Mature microRNAs (miRNAs) represent a kind of short, single-stranded, non-coding RNA, and they are identified as potential biomarkers in biomedical studies and clinical diagnoses. In cells, the mature microRNAs are produced from the cleavage of longer precursor microRNAs (pre-miRNAs, 60–70 nt) by RNase Dicer. As a standard method in vitro, quantitative real-time PCR (qRT-PCR) provides the ability to assess the abundance of mature miRNAs and pre-miRNAs in test tubes, but it is not easily applied to in situ biosensing of mature microRNA in living cells^60^. Although a number of intracellular miRNA biosensors have been developed based on the sequence complementarity of miRNAs and nucleic acid probes (e.g., molecular beacons), few can avoid the signal influence of pre-miRNA on mature microRNAs since the sequence of mature microRNAs is also present in the pre-miRNA^61,62^. Alternatively, since the length of mature microRNAs (19–23 nt) is much smaller than that of pre-miRNAs (60–70 nt), a size-selective sensing strategy based on differences in size is believed to be a promising method, while it has not been reported. Here, we employed size dependent permeable DNA nanocages to distinguish the short *mature miRNA-21* from the interference of longer *pre-miRNA-21* based on their difference in size. As shown in Fig. 6a, a CONFINED strategy-based molecular beacon (CONFINED-MB) with complementary sequence of *miRNA-21* was encapsulated in DNA cages. Since the molecular beacon encapsulated in Cage 2 (termed Cage-MB-in-2, Supplementary Fig. 28) exhibited better biosensing performance (Supplementary Fig. 29), we used the Cage-MB-in-2 for further experiments (named as Cage-MB-in). As a consequence of the MB’s hairpin structure, Cy3 fluorescence was quenched by BHQ-2. With the design of DNA Cage 2 based on its suitable cavity size, the mature *miRNA-21* could enter the cavity to hybridize with the MB and separate quencher from fluorophore, thereby triggering an increase of fluorescence signal. However, the *pre-miRNA-21* is too large to enter the cavity for hybridization with MB, thus showing negligible fluorescence change. The size-selective recognition of Cage-MB-in to distinguish different lengths of target nucleic acid sequences was first verified (Fig. 6b). The targets consist of the *miRNA-21* mimicry of sequences flanked by sequences with additional poly-T (named T10-miR21-T10, T20-miR21-T20, and T30-miR21-T30, respectively, as shown in (Supplementary Table 3). As shown in Fig. 6c and the fluorescence spectra of Supplementary Fig. 30a, the free MB probe without framework nucleic acids showed similar sensing performance to the different length of targets because all targets contained the sequence complementary to probe. However, Cage-MB-in probe exhibited stronger fluorescence response to target nucleic acids with decreasing total length (Fig. 6d and Supplementary Fig. 30b). These results confirmed the ability of Cage-MB-in to distinguish different lengths of target nucleic acids by size-selective recognition. In addition, the response kinetics of Cage-MB-in to target was proved to be similar to that of free MB probes (Supplementary Fig. 31) and still kept the responsive ability in the nuclease for longer time (Supplementary Fig. 32). Subsequently, we applied the size-selective recognition of Cage-MB-in to distinguish mature microRNA from pre-microRNA. As a negative control, the probe without cavity effect (Cage-MB-out) was also tested. As shown in Fig. 6e, f and Supplementary Fig. 33, the presence of *pre-miRNA-21* triggered an obvious signal increase in the case of Cage-MB-out (Fig. 6e), about 64% of the value for mature microRNA. However, the presence of *pre-miRNA-21* only induced 14% of the fluorescence increase in the Cage-MB-in probe (Fig. 6f), corresponding to about one fifth that of Cage-MB-out. Thus, Cage-MB-in significantly improved the sensing accuracy of MB. It also showed sensitive and selective detection ability for the mature microRNA in buffer (Supplementary Figs. 34 and 35) and human serum (Supplementary Fig. 36). Besides, the Cage-MB-in still kept the specifically recognition to *miRNA-21* in the mixed population of mature and precursor miRNAs (Supplementary Fig. 37). Furthermore, this method possessed the considerable quantitative ability compared with the qRT-PCR method (Supplementary Table 2).

### Intracellular size-selective discrimination

**Fig. 7:**
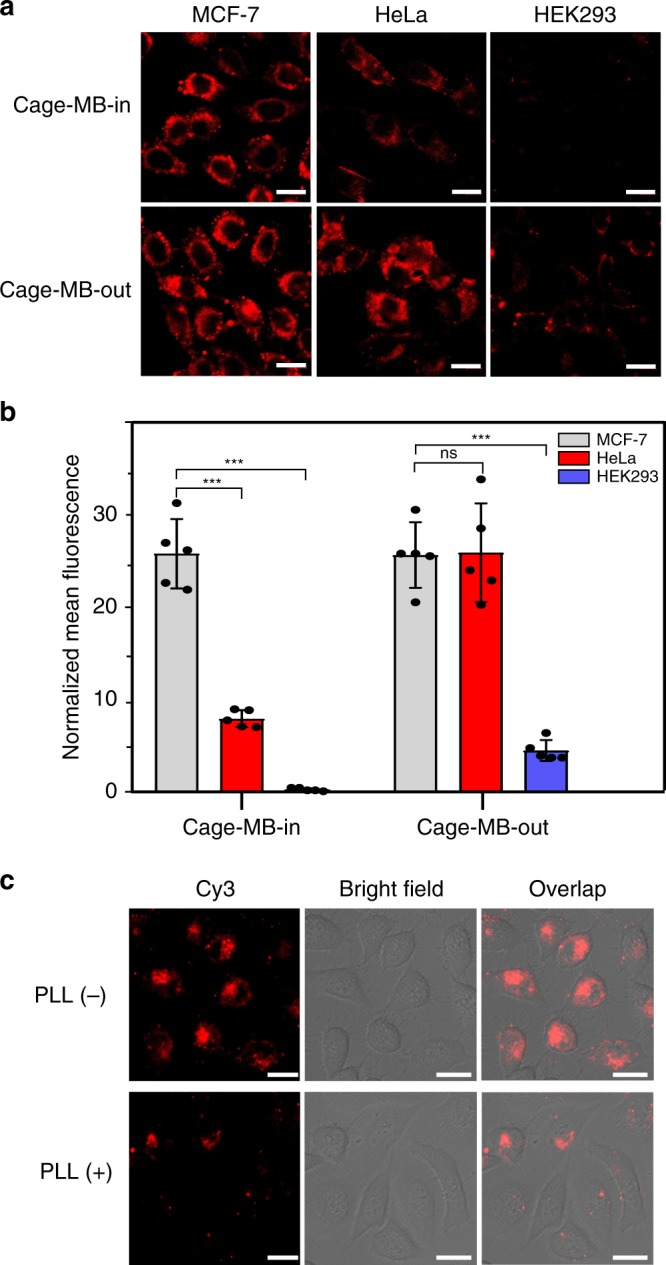
Size-selective discrimination between mature microRNA and precursor microRNA in living cells. **a**, **b** Fluorescence confocal imaging of MCF-7, HeLa and HEK293 cells incubated with 100 nM Cage-MB-in and Cage-MB-out (**a**) and the normalized mean fluorescence by ImageJ (**b**); Data are presented as mean values ± s.d. (*n* = 5), ***p = 0.00000068 (0.000000036), 0.0000014 for HeLa (HEK293) to Cage-MB-in, HEK293 to Cage-MB-out < 0.001, ns = 0.93 > 0.05 (not significant), by two-tailed unpaired Student’s *t*-test. **c** Fluorescence confocal imaging of HeLa cells treated with and without 5 μM *miRNA-21* inhibitor PLL before incubating with 100 nM Cage-in. The scale bars are 20 μm. Source data are provided as Source Data file.

The performance of Cage-MB-in for mature microRNA biosensing was further investigated in living cells. Three cancer cell lines, including MCF-7, HeLa and HEK293, with different *microRNA-21* expression levels were employed^8^. As control, Cage-MB-out was used under the same conditions. Results showed that HEK293 exhibited negligible fluorescence intensity, while MCF-7 showed a strong signal in the case of both Cage-MB-in and Cage-MB-out (Figs. 7a, b). Interestingly, the fluorescence intensity of HeLa cells was obviously lower than that of MCF-7 if Cage-MB-in probes were applied, but comparable to that of MCF-7 in the case of Cage-MB-out. To study the reason, the in vitro standard qRT-PCR method was employed to estimate the relative concentrations of *miRNA-21* and *pre-miRNA-21* in the three different cell lysate samples (Supplementary Fig. 38). Results showed that the abundance of mature *miRNA-21* of all three cell lines followed the order: MCF-7 > HeLa> HEK293, which is closer to the results for Cage-MB-in rather than the results for Cage-MB-out (Fig. 7c). The results indicated that Cage-MB-out failed to accurately sense the abundance of mature *miRNA-21* because of the false signal produced from *pre-miRNA-21*. On the contrary, Cage-MB-in could successfully distinguish the signal of mature *miRNA-21* from the interference of *pre-miRNA-21*, again suggesting the excellent size-selective sensing ability of the DNA molecular sieve. Finally, Cage-MB-in was applied to measure the change in abundance of *miRNA-21* during drug treatment. As an inhibitor of Dicer activity^63^, poly-l-lysine (PLL) can reduce the production of mature *miRNA-21*, which was validated by qRT-PCR (Supplementary Fig. S39). As shown in Fig. 7c, the fluorescence signal of HeLa cells significantly decreased after treatment with PLL, indicating the decrease of mature *miRNA-21* abundance. This result agreed with the previous report and supported the potential of Cage-MB-in for distinguishing mature microRNA from its precursor in living cells.

## Discussion

In summary, we have demonstrated a DNA sieve for size-selective molecular recognition by encapsulating molecular recognition elements (e.g. FNAs) into a cavity-tunable framework nucleic acid. The DNA molecular sieve-based size-selective molecular recognition system exhibited several advantages. First, benefiting from the protection of framework nucleic acids, the FNAs exhibited enhanced anti-interference ability against nuclease degradation and nonspecific protein binding, thus providing FNAs with high stability and accuracy for intracellular sensing. Second, the programmability and predictability of DNA nanotechnology provided a promising platform for the construction of size-selective sensing system. For example, the size-selective discrimination between intracellular mature microRNA and its precursor was demonstrated in this work. Third, framework nucleic acids are easy to design and prepare; they also show better biocompatibility compared to inorganic materials. With these features, the DNA molecular sieve achieved the size-selective and accurate sensing of a wide range of biological targets in a precise manner. Although the detection sensitivity of current probes is unsatisfactory for some specific situations, it could be further improved for sensitive biosensing in real samples by combining this strategy with some efficient signal amplification strategies such as hybridization chain reaction^1^. Therefore, the strategy of controlling the cavity of framework nucleic acids for DNA molecular sieve is expected to be applied for the molecular recognition of other important biological targets with different sizes, such as the monomer and dimer during proteins dimerization process, thus providing a blueprint for size-selective molecular recognition in biomedical studies and clinical diagnoses.

## Methods

### Materials and reagents

All DNA oligonucleotides (Supplementary Table 3 in Supporting Information) were purchased from Sangon Biotech Co. Ltd. (Shanghai, China). The microRNA sequences, Hairpin-it^TM^ microRNA and U6 snRNA Normalization RT-PCR Quantitation Kit were purchased from Shanghai GenePharma (Shanghai, China). Eastep® Super Total RNA Extraction Kit was purchased from Promega (Madison, WI). DNase I, Single-strand binding protein (SSB) were purchased from Takara Biotechnology Co.Ltd. (Dalian, China). l-histidine and their respective analogues were obtained from Sigma-Aldrich (Milwaukee, WI, USA). Adenosine triphosphate (ATP) and poly-l-lysine (PLL) were obtained from Sigma-Aldrich (Milwaukee, WI, USA). Lipofectamine 3000 was purchased from Thermo-Fisher Scientific (Pittsburgh, PA, USA). Other reagents were all of analytical grade and used without further treatment. Ultrapure water was obtained from a Milli-Q system (Billerica, MA, USA). The fluorescence measurements were performed on a Fluoromax-4 (HORIBA Jobin Yvon Inc., Edison, NJ) spectrofluorometer at room temperature. The AFM characterization of the sample was carried out on a Bruker Multimode V8 Scanning Probe Microscope (Bruker, German). The confocal fluorescence imaging studies were performed on a FV1000 confocal laser-scanning microscope (Olympus, Tokyo, Japan) & Zeiss LSM 880 (Carl Zeiss, Jena, Germany). MTS assay was performed with a Synergy 2 Multi-Mode Microplate Reader (Bio-Tek, Winooski, VT). All cell lines were purchased from ATCC. We collected human blood serum from healthy volunteers.

### Assembly of DNA nanocages

The equal molar ratio of customized single-stranded oligonucleotide strands (1 μM for page fluorescence, 100 nM for fluorescence analysis) were mixed in the tris-acetic acid-magnesium (TAE-Mg^2+^) buffer (20 mM Tris, 2 mM EDTA, 12.5 mM MgCl_2_, pH 7.4) and annealed from 95 to 4 °C over 6.5 h. All these sequences were listed in Supplementary Table 3.

### Native-PAGE analysis

For Cage 1 and Cage 2, we used 8% native polyacrylamide gel electrophoresis with 1xTAE-Mg^2+^ with 110 V in an ice-water bath for 2 h for characterization. For Cage 3 to Cage 5, we used 5% native-PAGE for characterization. All results were analyzed by a fluorescence image scanner (FLA-3000G, Fuji, Tokyo, Japan).

### Fluorescence analysis

The fluorescence spectrum was determined using Fluoromax-4 fluorescence spectrometer. The dye of DNAzyme and ATP aptamer is FAM, which was excited at 488 nm, and the fluorescence emission spectra was recorded from 505 to 650 nm. The dye of molecular beacon for *micro RNA-21* is Cy3, which was excited at 525 nm, and the fluorescence emission spectra was recorded from 545 to 650 nm. The concentration of DNA nanocages used for the fluorescence calibration curve assays was 10 nM (probe: DNA nanocage = 1:1) in HEPES buffer (25 mM HEPES, 500 mM KCl, pH 7.9) and TAE-Mg^2+^ buffer for l-histidine and ATP detection, respectively. The concentration of DNA nanocages for microRNA detection is 20 nM (probe: DNA nanocage = 1:1). Then, the targets at a series of concentrations were incubated with DNA cage for 30 min at 37 °C. For the DNAzyme selectivity experiments, the concentrations of other conditions of control groups were ten times of that of l-histidine. For Cage-apt and Cage-micro, the concentration and other conditions of control groups were same as that of the experiment group. For the measurement of *microRNA-21* in human blood serum, the Cage-MB-in-2 (20 nM) incubated with different concentrations of *microRNA-21* mimics in the 100% human serum for 1 h, and then determined by Fluoromax-4 fluorescence spectrometer.

### Stability experiment

The concentrations of probes were 10 nM. In nuclease stability experiments, the fluorescence change of all samples was used time-scan model under the treatment of DNase I enzyme (1 U mL^−1^). In protein binding experiments, the fluorescence change of all samples was used the fluorescence spectral scanning model under the treatment of single-strand binding protein (SSB, 40 nM).

### AFM imaging

In all, 2 µL of sample (5 nM) was dropped on a freshly cleaved mica surface (Ted Pella, Inc.). After 2 min, the sample was mixed with 50 µL 1xTAE-Mg^2+^ buffer and 2 µL 100 mM Ni^2+^. The samples were scanned with SCANASYST-FLUID probes (Bruker) in “ScanAsyst in fluid” mode using a Bruker Multimode V8 Scanning Probe Microscope (Bruker, Germany).

### DLS characterization

Samples (500 µL) of DNA nanocages (500 nM) were prepared for dynamic light scattering analysis. DNase I and SSB were measured with a concentration of 1 mg mL^−1^.

### Preparation of cell lysates

The cell lysates were prepared by breast cancer HeLa cells which cultured in Dulbecco’s modified Eagle’s medium (DMEM) with 10% inactivated fetal bovine serum and 1% penicillin in 5% CO_2_ at 37 °C. First, the cell were plated in 35 mm cell culture dish and cultured to ~80% before experiment. Cells were washed with 1 mL DPBS, and 200 µL of trypsin was then added to the cell culture dish for 3 min. After that, HeLa cells were centrifuged for 3 min at 25 °C (1000* × g*), the supernatant fraction was removed, and the cell re-dispersed in a 1 mL of buffer solution. Then, the re-dispersed cells were treated with sonication treatment in ice-water bath using a probe-type sonicator (200 W).

### MTS assay

The cytotoxicity of DNA nanocages to cells was conducted by MTS analysis. First, the cells were seeded in a 96-well plate for 24 h with 2 × 10^5^ cells per well. After removing cell medium, cells were first incubated with different concentrations of DNA nanocages (0, 20, 40, 60, 120, and 240 nM) in washing buffer (DPBS buffer containing 5 mM MgCl_2_ and 4.5 mg mL^−1^ glucose) at 37 °C for 4 h to allow sufficient uptake. At the preassigned time, the cell medium was replaced with 200 µL of fresh cell medium. After 48 h incubation, the cell medium was replaced with 100 µL of fresh cell medium containing 20 µL of MTS solution (Promega) to each well. After 30 min incubation, cell viability was measured via the absorbance at 490 nm by a Synergy 2 Multi-Mode Microplate Reader (Bio-Tek, Winooski, VT).

### Intracellular imaging

MCF-7, HeLa and HEK293 cell lines (from ATCC) were grown in Dulbecco’s modified Eagle’s medium (DMEM) with 10% inactivated fetal bovine serum and 1% penicillin in 5% CO_2_ at 37 °C. For l-histidine imaging, HeLa cells were first incubated with Czyme-in-2 or free DNAzyme in DMEM for 4 h, followed by washing with PBS and treatment with or without 100 mM of l-histidine for another 1 h. Finally, the confocal fluorescence imaging of each dish was carried out on the FV1000 confocal laser-scanning microscope. The fluorescence signal was recorded from 500 to 540 nm with 488 nm laser excitation. For intracellular ATP imaging, the HeLa cell lines were cultured in DMEM medium with 10% inactivated fetal bovine serum and 1% penicillin for 24 h. Before the addition of Cage-apt, the cells were incubated with DPBS buffer, 10 µg mL^−1^ oligomycin or 100 µM etoposide for 0.5 h, respectively. Then, the Cage-apt probes were incubated with cells for another 4 h, followed by washing three times and fluorescent confocal imaging. The imaging results were analyzed using ImageJ. For confocal imaging of *miRNA-21*, the HeLa, MCF-7 and HEK293 cell lines were cultured in DMEM medium with 10% inactivated fetal bovine serum and 1% penicillin for 24 h, and then Cage-MB-in probes were incubated with cells for another 4 h, followed by washing three times and fluorescent confocal imaging. The fluorescence signal was recorded from 548 nm to 681 nm with 543 nm laser excitation. The expression levels of *miRNA-21* and *pre-miRNA-21* in living cells were regulated by pretreated with 5 µM poly-l-lysine inhibitor for two days.

### Liposome transfection

HeLa cells were seeded in a glass-bottomed dish and incubated for 24 h. Before transfection, 25 µL of Opti-MEM® medium containing free DNAzyme was added to a 1.5 mL tube, and 25 µL Opti-MEM® medium containing 1 µL Lipofectamine-3000 was added to another 1.5 mL tube. After briefly mixing the contents of the two tubes, the sample (100 nM) was incubated at 25 °C for 10–15 min. At the preassigned time, the liposome complex was diluted to 100 µL and then added to the cells.

### ATP measurement using UV-vis absorption spectrometry

In order to acquire the standard curve of ATP by UV-vis absorption spectrometry (UV-2600, Shimadzu, Japan), we diluted the sample at the final concentration of 100, 200, 300, 400, 500, and 600 µM respectively, and measured their absorption at 260 nm.

### qRT-PCR Analysis

Total RNAs from MCF-7, HeLa and HEK293 cells were extracted using the Eastep® Super Total RNA Extraction Kit following the manufacturer’s instructions. The extracted RNA was re-dispersed in 30 µL RNase-free water. The cDNA samples were prepared using the reverse transcription (RT) reaction of Hairpin-it^TM^ Real-Time PCR kit purchased from GenePharma (Shanghai, China) according to the user manual. For *miRNA-21* and *pre-miRNA-21* analysis, the cDNA was prepared using the corresponding microRNA-21 or pre-microRNA U6 snRNA RT primer mix supplied by GenePharma. The qRT-PCR analysis of miRNA and pre-microRNA was performed using 2 × Real-time qPCR Master Mix (SYBR) with ROX on an Applied Biosystem 7000 (USA). The primers (from 5′ to 3′) used in this experiment (supplied by GenePharma) are listed below. We evaluated all the data with respect to miRNA and pre-miRNA expression by normalizing to the expression of U6 and using the 2^−∆∆Ct^ method^64^. All primer sequences were listed in Supplementary Table 3.

### Statistical analysis

All numerical data, AFM imaging data, fluorescence imaging data of living cells are collected from a minimum of three independent experiments unless otherwise specified. No data were excluded in the studies. Numerical data are presented as mean values ± s.d. Student’s *t*-test was applied for comparison of two groups by Microsoft excel 2013’s statistical tools (T.TEST) to assess significance (*P* value). Fluorescence spectral data and UV absorbance data were analyzed with the Origin Lab 2017 and GraphPad Prism 8.0.1. Statistical mean and differences were evaluated using Microsoft excel 2013’s statistical tools and the GraphPad Prism 8.0.1. The gel image data were analyzed with the Bio-Rad ChemiDoc XRS System. Confocal imaging data was analyzed using the Carl Zeiss ZEN 2 (blue edition), Olympus Analysis Software and ImageJ.

### Reporting summary

Further information on research design is available in the Nature Research Reporting Summary linked to this article.

## Supplementary information

Supplementary Information

Reporting Summary

## Data Availability

The main data in this work are available in the main manuscript and Supplementary Information. The source data underlying Figs. 3b, 4c, f, 5b, d, 6c–f, 7b and Supplementary Figs. 11, 16b, 17, 19, 20, 22, 23, 26, 27, 29, 33, 35, 37, 38, 39 and Tables S1, S2 are provided as a Source Data file. Additional data are available from the corresponding author upon reasonable request.
