# Supplementary material for: Size-selective molecular recognition based on a confined DNA molecular sieve using cavity-tunable framework nucleic acids
Source: Nat Commun. 2020 Mar 23;11:1518. doi: 10.1038/s41467-020-15297-7 (PMC7089997; doi:10.1038/s41467-020-15297-7)
Supplement: Supplementary file 1 — Supplementary Information [file 41467_2020_15297_MOESM1_ESM.pdf]

Supplementary Information

for

Size-selective Molecular Recognition based on a Confined DNA Molecular Sieve using Cavity-tunable Framework Nucleic Acids

Fu, Ke *et al.*

Supplementary Figures

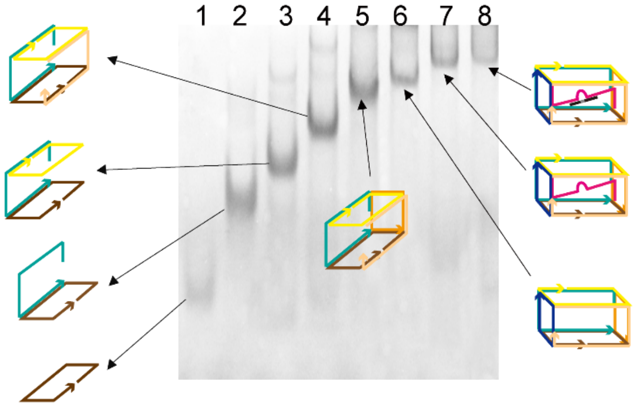

Lane 1: C1-2;
Lane 2: C1-2+C1-3;
Lane 3: C1-2+C1-3+C1-5;
Lane 4: C1-2+C1-3+C1-5+C1-6;
Lane 5: C1-2+C1-3+C1-5+C1-6+C2-4;
Lane 6: C1-2+C1-3+C1-5+C1-6+C2-4+C2-1;
Lane 7: C1-2+C1-3+C1-5+C1-6+C2-4+C2-1+C-DNAzyme-DABCLY;
Lane 8: C1-2+C1-3+C1-5+C1-6+C2-4+C2-1+C-DNAzyme-DABCLY +C-d-FAM-DABCLY.

Supplementary Figure 1 | Native-PAGE analysis of Cage 1. 8% native-PAGE analysis of the stepwise assembly of Cage 1 (lane 1 to lane 6) and the hybridization of the double stranded DNAzyme cargo (lane 7 and lane 8). (The concentration of each strand is 1 μM).

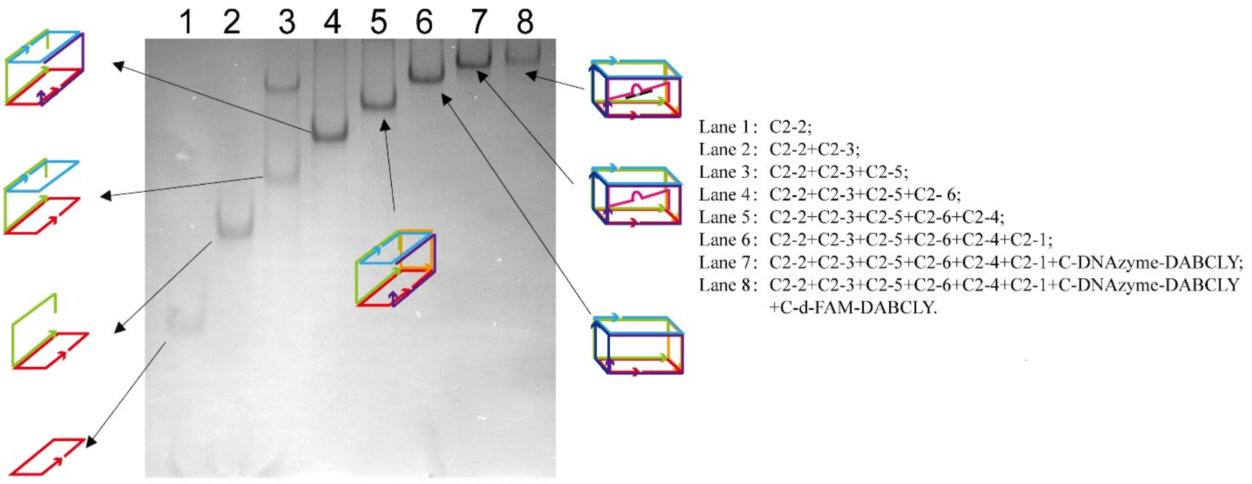

Supplementary Figure 2 | Native-PAGE analysis of Cage 2. 8% native-PAGE analysis of the stepwise assembly of Cage 2 (lane 1 to lane 6) and the hybridization of the double-stranded DNAzyme cargo (lane 7 and lane 8). (The concentration of each strand is 1 μM).

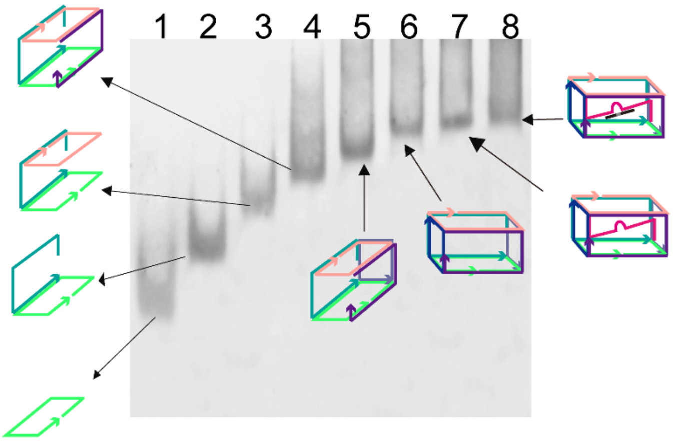

Lane 1: C3-2;
Lane 2: C3-2+C2-3;
Lane 3: C3-2+C2-3+C3-5;
Lane 4: C3-2+C2-3+C3-5+C2-6;
Lane 5: C3-2+C2-3+C3-5+C2-6+C3-4;
Lane 6: C3-2+C2-3+C3-5+C2-6+C3-4+C3-1;
Lane 7: C3-2+C2-3+C3-5+C2-6+C3-4+C3-1+C-DNAzyme-DABCLY;
Lane 8: C3-2+C2-3+C3-5+C2-6+C3-4+C3-1+C-DNAzyme-DABCLY +C-d-FAM-DABCLY.

Supplementary Figure 3 | Native-PAGE analysis of Cage 3. 5% native-PAGE analysis of the stepwise assembly of Cage 3 (lane 1 to lane 6) and the hybridization of the double-stranded DNAzyme cargo (lane 7 and lane 8). (The concentration of each strand is 1 μM).

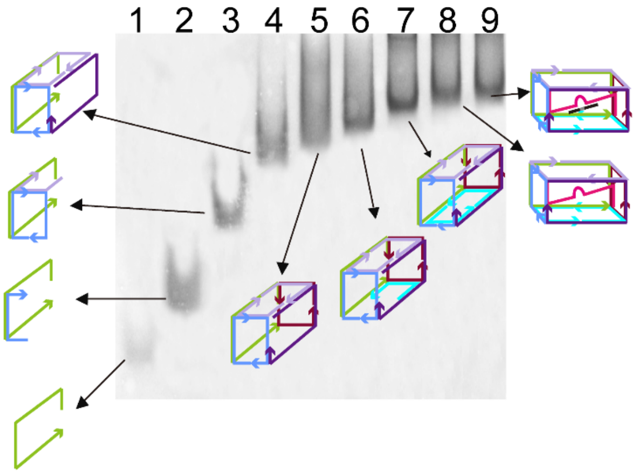

Lane 1: C2-3;
Lane 2: C2-3+C4-1-2;
Lane 3: C2-3+C4-1-2+C4-1-1+C4-5-2;
Lane 4: C2-3+C4-1-2+C4-1-1+C4-5-2+C4-5-1+C2-6;
Lane 5: C2-3+C4-1-2+C4-1-1+C4-5-2+C4-5-1+C2-6+C4-4-1+C4-4-2;
Lane 6: C2-3+C4-1-2+C4-1-1+C4-5-2+C4-5-1+C2-6+C4-4-1+C4-4-2 +C4-2-1;
Lane 7: C2-3+C4-1-2+C4-1-1+C4-5-2+C4-5-1+C2-6+C4-4-1+C4-4-2 +C4-2-1+C4-2-2;
Lane 8: C2-3+C4-1-2+C4-1-1+C4-5-2+C4-5-1+C2-6+C4-4-1+C4-4-2 +C4-2-1+C4-2-2+C-DNAzyme-DABCLY;
Lane 9:C2-3+C4-1-2+C4-1-1+C4-5-2+C4-5-1+C2-6+C4-4-1+C4-4-2 +C4-2-1+C4-2-2+C-DNAzyme-DABCLY+C-d-FAM- DABCLY.

Supplementary Figure 4 | Native-PAGE analysis of Cage 4. 5% native-PAGE analysis of the stepwise assembly of Cage 4 (lane 1 to lane 7) and the hybridization of the double-stranded DNAzyme cargo (lane 8 and lane 9). (The concentration of each strand is 1 μM).

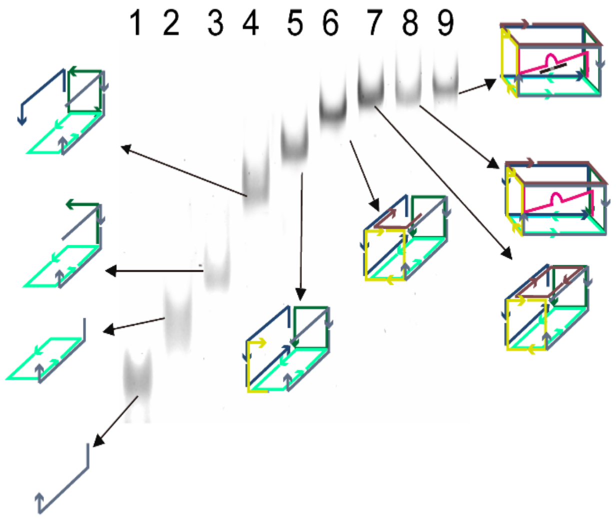

Lane 1: C5-6-2;
Lane 2: C5-6-2+C5-2-2+C5-2-1;
Lane 3: C5-6-2+C5-2-2+C5-2-1+C5-6-1+C5-4-1;
Lane 4: C5-6-2+C5-2-2+C5-2-1+C5-6-1+C5-4-1+C5-4-2+C5-3-1;
Lane 5: C5-6-2+C5-2-2+C5-2-1+C5-6-1+C5-4-1+C5-4-2+C5-3-1+C5-3-2 +C5-1-2;
Lane 6: C5-6-2+C5-2-2+C5-2-1+C5-6-1+C5-4-1+C5-4-2+C5-3-1+C5-3-2 +C5-1-2+C5-1-1+C5-5-2;
Lane 7: C5-6-2+C5-2-2+C5-2-1+C5-6-1+C5-4-1+C5-4-2+C5-3-1+C5-3-2 +C5-1-2+C5-1-1+C5-5-2+C5-5-1;
Lane 8: C5-6-2+C5-2-2+C5-2-1+C5-6-1+C5-4-1+C5-4-2+C5-3-1+C5-3-2 +C5-1-2+C5-1-1+C5-5-2+C5-5-1+C-DNAzyme-DABCLY;
Lane 9: C5-6-2+C5-2-2+C5-2-1+C5-6-1+C5-4-1+C5-4-2+C5-3-1+C5-3-2 +C5-1-2+C5-1-1+C5-5-2+C5-5-1+C-DNAzyme-DABCLY+C-d-FAM-DABCLY.

Supplementary Figure 5 | Native-PAGE analysis of Cage 5. 5% native-PAGE analysis of the stepwise assembly of Cage 5 (lane 1 to lane 7) and the hybridization of the double-stranded DNAzyme cargo (lane 8 and lane 9). (The concentration of each strand is 1 μM).

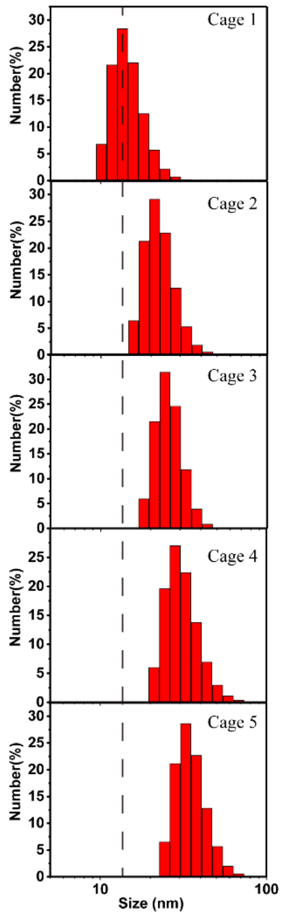

Supplementary Figure 6 | DLS of Cage 1 - Cage 5. The results indicated a gradual increase of the hydration radius of DNA nanocages from cage 1 to cage 5.

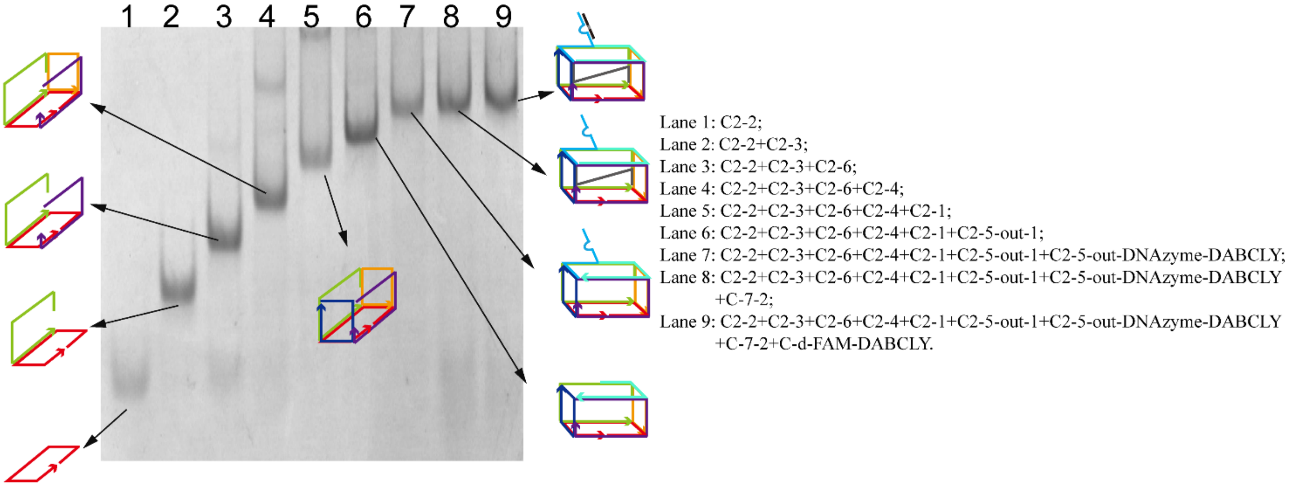

Supplementary Figure 7 | Native-PAGE analysis of Czyme-out-2. PAGE analysis of the Czyme-out-2 stepwise assembly by 8% native-PAGE. (The concentration of each strand is 1 μM).

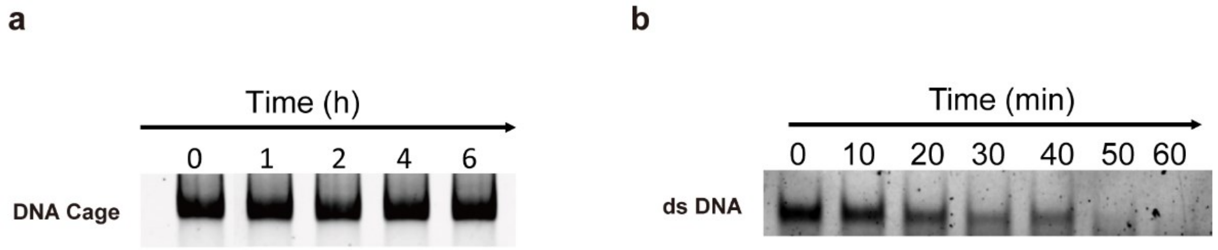

Supplementary Figure 8 | The stability of DNA Cage in DNase I. **a** The native-PAGE analysis of DNA Cage (50 nM) after treated with 1 U mL^{-1} DNase I in different incubation time. **b** The PAGE of double-stranded DNA probe (ds DNA) after treated with 1 U mL^{-1} DNase I in incubation time. As the results shown, the DNA cage still kept stable in 1 U mL^{-1} DNase I for longer time point, however the double-stranded DNA (ds DNA) was gradually digested by DNase I within 1 hour.

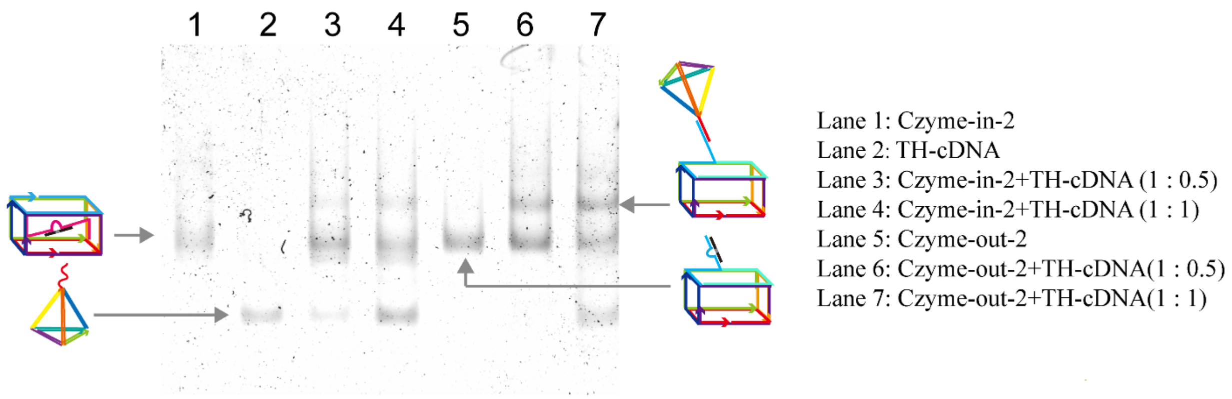

Supplementary Figure 9 | Encapsulated performance of Czyme-in-2. 5% native-PAGE analysis confirmed the addressable modification of DNAzyme inside and outside the cavity of DNA nanocage in Czyme-in-2 and Czyme-out-2, respectively. In this experiment, a DNA tetrahedron-tailed cDNA (TH-cDNA) with complementary sequence to the loop sequence of DNAzyme was incubated with Czyme-in-2 and Czyme-out-2, respectively, followed a native-PAGE analysis. Since DNA tetrahedron is too large to enter the cavity, TH-cDNA is expected to hybridize with the loop of DNAzyme outside the cavity rather than inside the cavity. As except, the PAGE result showed that TH-cDNA hybridized with Czyme-out-2 (indicated by the obvious upper new band), while didn't bind with Czyme-in-2 (no obvious upper new band appeared). These results suggested the successful binding of DNAzyme inside and outside the cavity in Czyme-in-2 and Czyme-out-2, respectively.

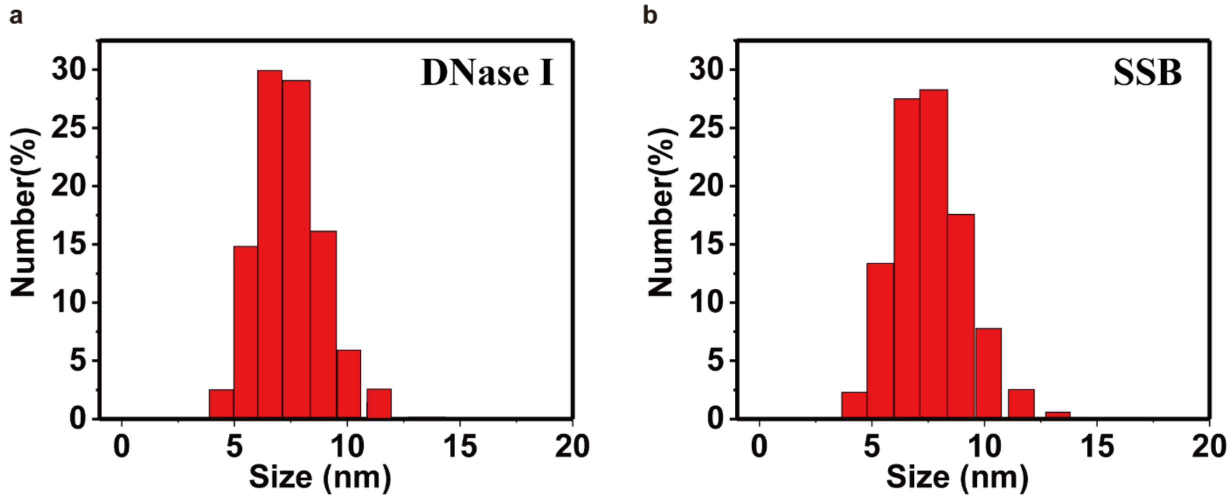

Supplementary Figure 10 | Size characterization of DNase. Determination of the size of (**a**) DNase I and (**b**) SSB using DLS.

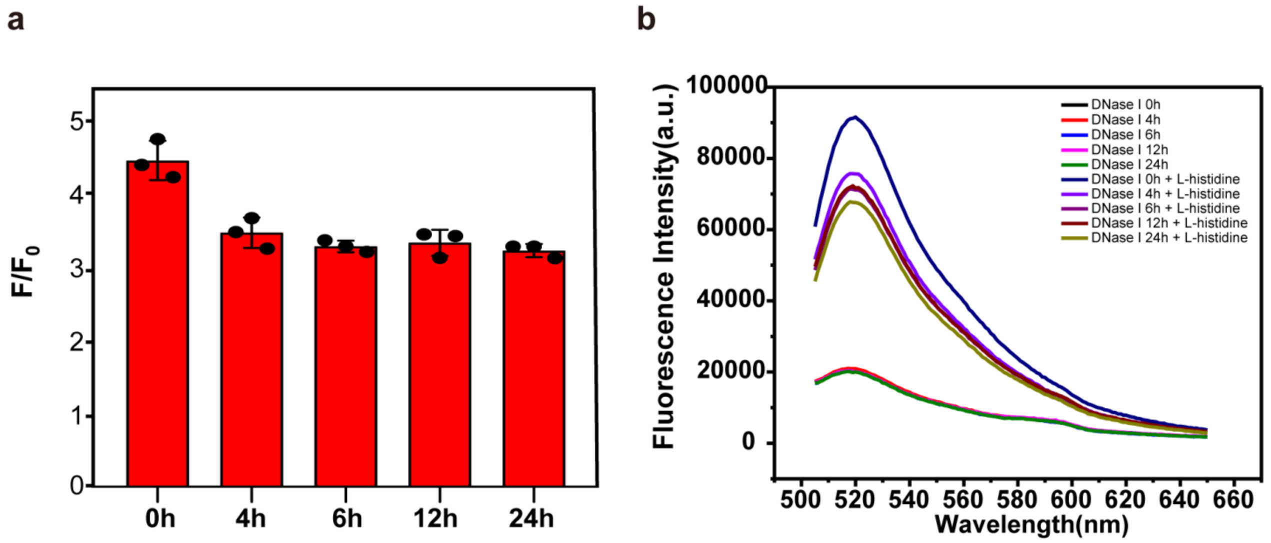

Supplementary Figure 11 | The response ability of Czyme-in-2 to L-histidine after treated with 0.25 U mL^{-1} DNase I at different time points. **a** The fluorescence ratio of Czyme-in-2 with (F) and without (F_0) target L-histidine in DNase I for different time point; Data are presented as mean values ± s.d. (n = 3). **b** The fluorescence spectra corresponding to **a**.

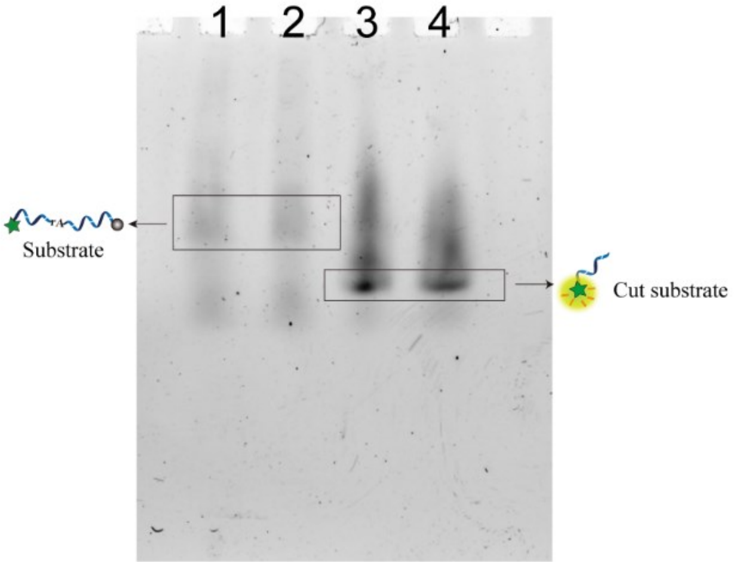

Supplementary Figure 12 | Responsive analysis of Czyme-in-2 to L-histidine by 10% denaturing PAGE. Line 1, Czyme-in-2 (500 nM); line 2, Cyzme-in-2 (500 nM) treated with 1 U mL^{-1} DNase I for 6 h; line 3, Czyme-in-2 catalytic cutting of substrates in the presence of L-histidine; line 4, Czyme-in-2 catalytic cutting of substrates in the presence of L-histidine after treated with 1 U mL^{-1} DNase I for 6 h. As the results shown, compared the Lane 1-2 with 3-4, the substrate strand band is upper and darker than the substrate strand cut by the DNAzyme in the absence of target. Once in the presence of target, the substrate strand was digested into two short strands that separated the fluorophore and quencher on the two ends of substrate, thus generating brighter bands that below the un-digested strand. Furthermore, the Czyme-in-2 still kept the similar responsive ability with (lane 2, 4) and without (Lane 1, 3) DNase I treated.

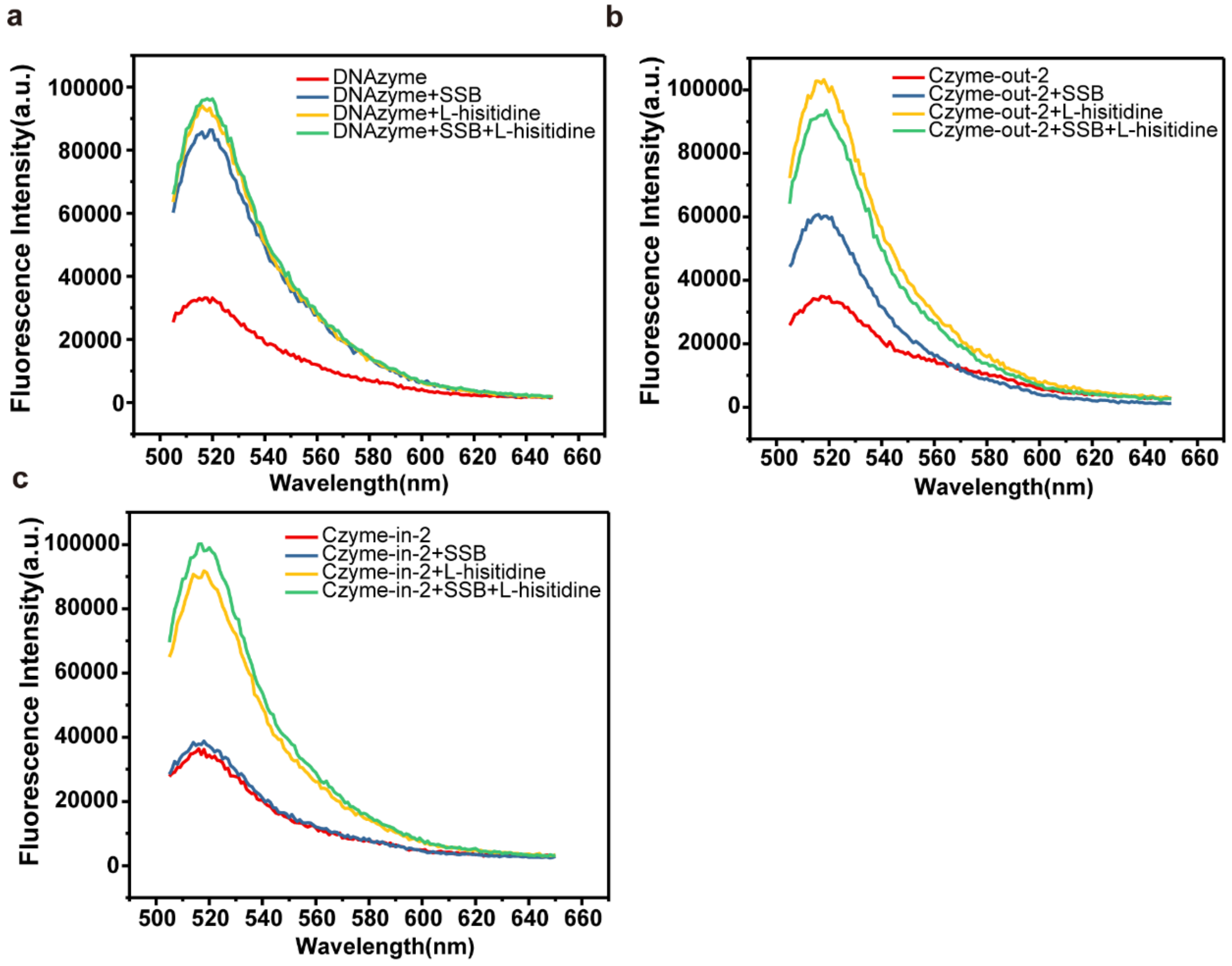

Supplementary Figure 13 | Fluorescence spectral of free DNAzyme, Czyme-out-2 and Czyme-in-2 to L-histidine treated with and without SSB. a Fluorescence responsive of DNAzyme to L-histidine with and without SSB. **b** Fluorescence responsive of Czyme-out-2 to L-histidine with and without SSB. **c** Fluorescence responsive of Czyme-in-2 to L-histidine with and without SSB.

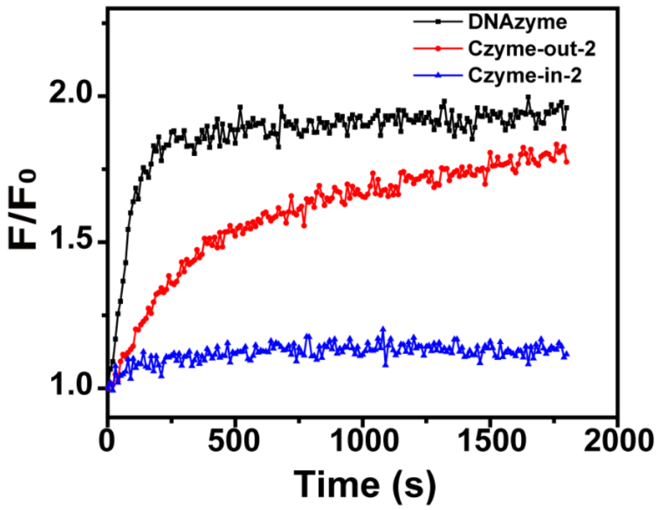

Supplementary Figure 14 | Cell lysate analysis. Stability of free DNAzyme, Czyme-out-2 and Czyme-in-2 in the cell lysate.

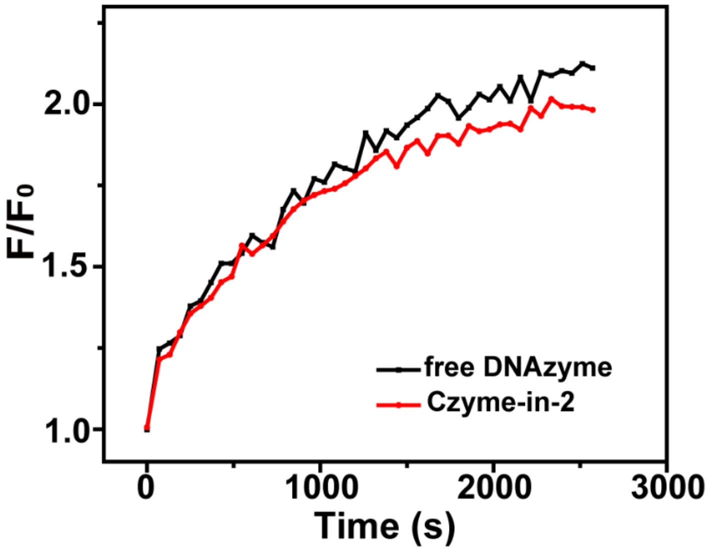

Supplementary Figure 15 | Sensing performance of Czyme-in-2. Comparison of response kinetics between free DNAzyme and Czyme-in-2 (C-DNAzyme-DABCLY: C-d-FAM-DABCLY = 1:1) in the presence of L-histidine.

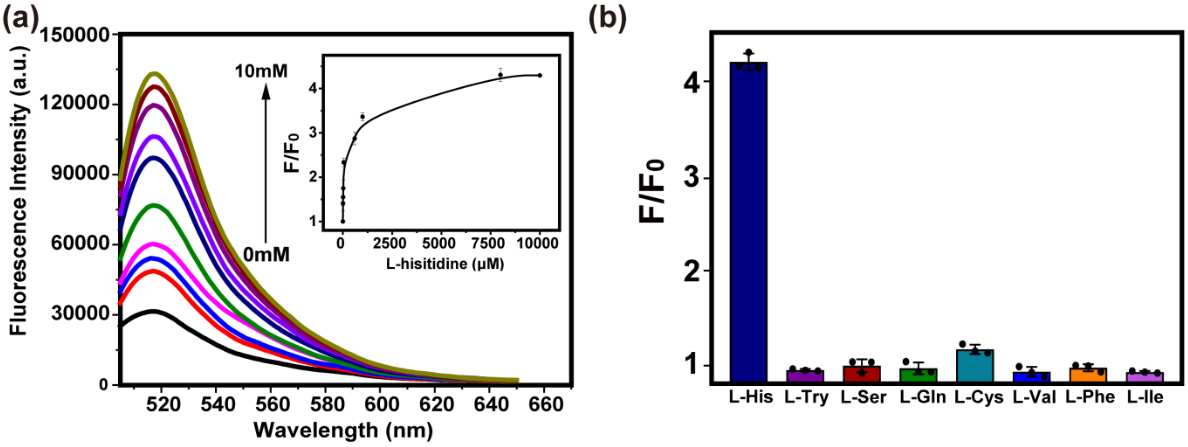

Supplementary Figure 16 | Sensing performance of Czyme-in-2 (C-DNAzyme-DABCLY: C-d-FAM-DABCLY= 2:1). a Fluorescence response of Czyme-in-2 in the presence of different concentrations of L-histidine, ranging from 0 to 10 mM. Inset: relationship between fluorescence enhancement and L-histidine concentrations (the detection limit was estimated to be 2.9 μM in terms of the rule of 3 times the standard deviation divided by the blank response). **b** Selectivity studies of Czyme-in-2. Concentration of L-histidine and other amino acids were 10 mM and 100 mM, respectively. Data are presented as mean values ± s.d. (n = 3).

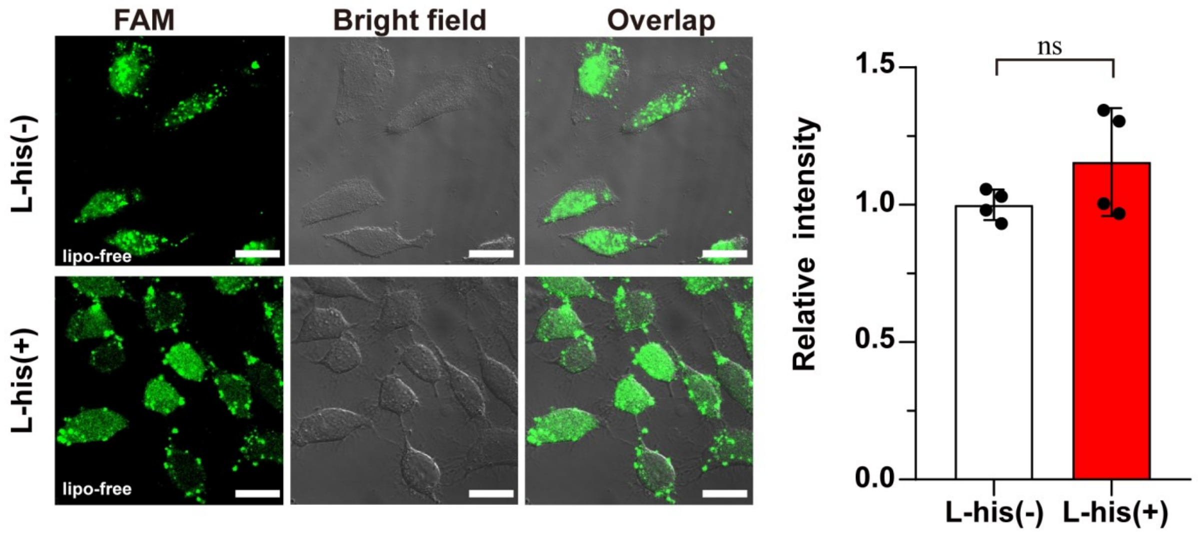

Supplementary Figure 17 | Fluorescent confocal imaging of free DNAzyme. Fluorescent confocal imaging of HeLa cells transfected with free DNAzyme by liposome (named lipo-free) with (L-his (+)) and without L-histidine (L-his (-)). The scale bars are 20 μm. The right graph is the relative fluorescent intensity quantified by Image J; Data are presented as mean values ± s.d. (n=4); ns = 0.18 > 0.05 (not significant), by two-tailed unpaired Student's *t*-test. The free DNAzyme showed negligible fluorescence enhancement in the presence of L-histidine owing to the high background signal due to the degradation of probes.

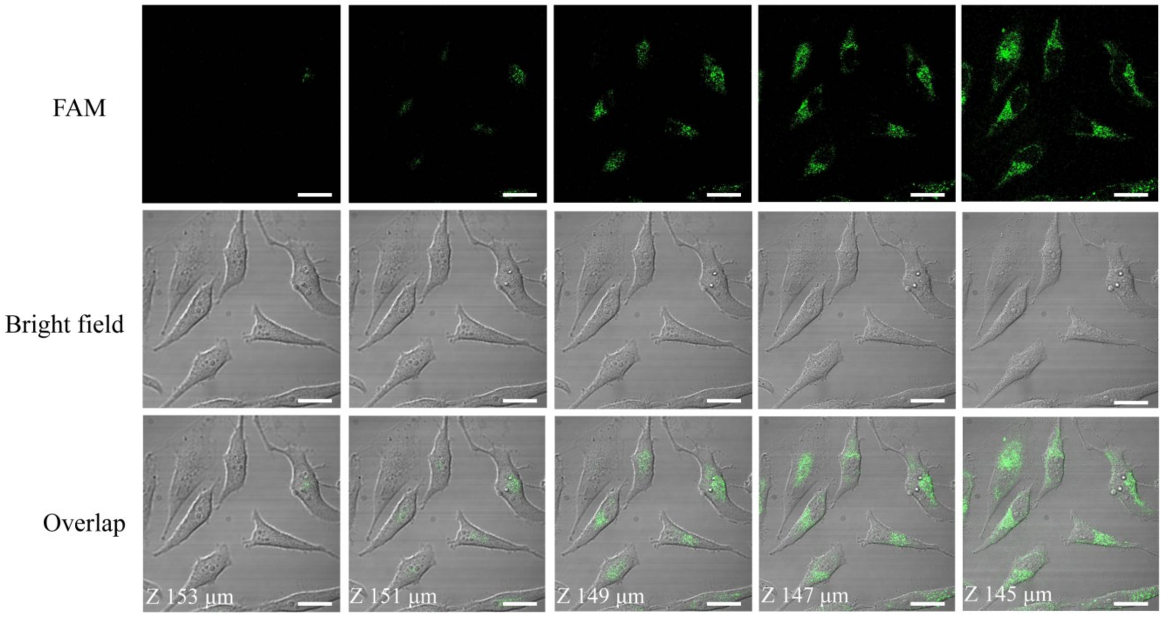

Supplementary Figure 18 | Z-axis scanning images. The z-axis scanning images of HeLa cells treated with Czyme-in-2 and L-histidine. The scale bars are 20 μm.

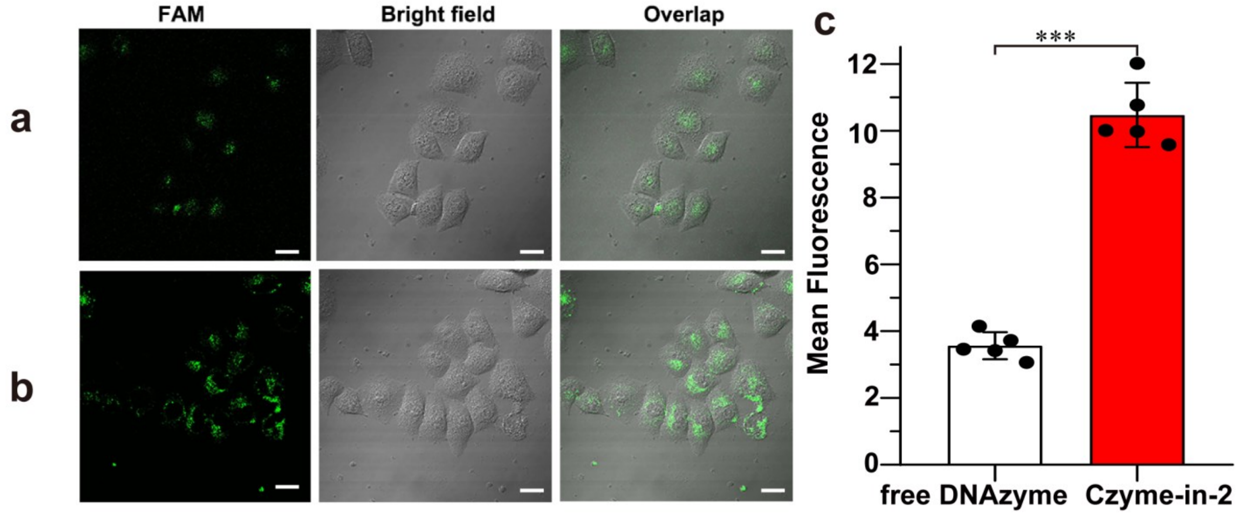

Supplementary Figure 19 | Fluorescent confocal imaging of free DNAzyme and Czyme-in-2. a-b Confocal microscopy fluorescence (left), bright field (middle), and overlay (right) images of cells after incubation with free DNAzyme (**a**) or Czyme-in-2 (**b**) in the presence of L-histidine. The scale bars are 20 μm. **c** Semiquantitative analysis of fluorescence intensity of free DNAzyme and Czyme-in-2 in cells by Image J. Data are presented as mean values ± s.d. (n = 5); ***p = 0.00000043 < 0.001, by two-tailed unpaired Student's t-test. The result showed that Czyme-in-2 could enter cells without transfection and was stable to response to histidine.

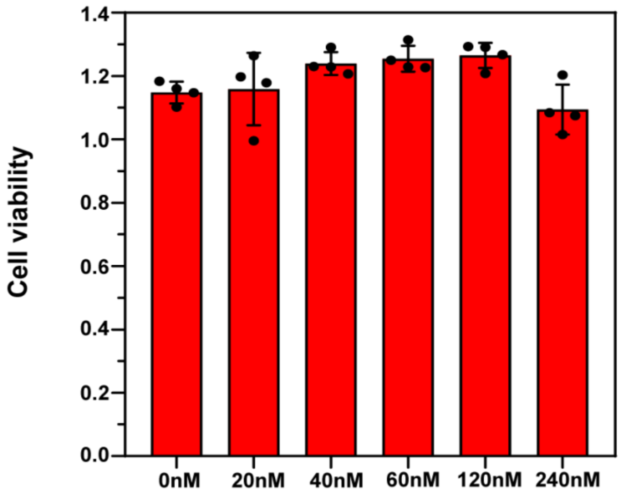

Supplementary Figure 20 | Cell viability. MTS assays of Czyme-in-2 with different concentration; Data are presented as mean values ± s.d. (n = 4).

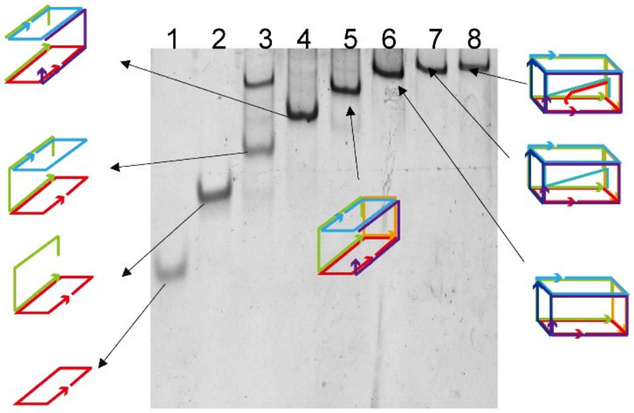

Lane 1 : C2-2
Lane 2 : C2-2+C2-3
Lane 3 : C2-2+C2-3+C2-5
Lane 4 : C2-2+C2-3+C2-5+C2- 6
Lane 5 : C2-2+C2-3+C2-5+C2-6+C2-4
Lane 6 : C2-2+C2-3+C2-5+C2-6+C2-4+C2-1
Lane 7 : C2-2+C2-3+C2-5+C2-6+C2-4+C2-1+C-cDNA
Lane 8 : C2-2+C2-3+C2-5+C2-6+C2-4+C2-1+C-cDNA+ATP aptamer

Supplementary Figure 21 | Native-PAGE analysis of Cage-apt. Analysis of the Cage-apt-in-2 assembly by 8% native-PAGE (the concentration of each strand is 1 μM).

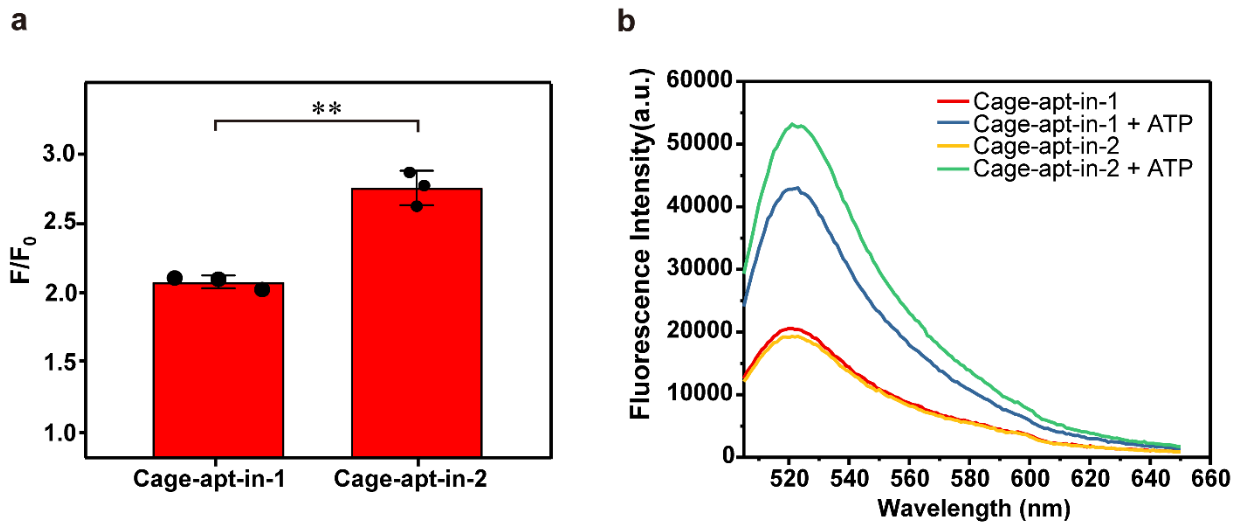

Supplementary Figure 22 | Comparison of biosensing ability between Cage-apt-in-1 and Cage-apt-in-2. a The fluorescence ratio of Cage-apt-in-1 and Cage-apt-in-2 (10 nM) with (F) and without (F_0) target ATP (1 mM), Data are presented as mean values ± s.d. (n=3); $**p = 0.0012 < 0.01$, by two-tailed unpaired Student's *t*-test. **b** The fluorescence spectra corresponding to **a**.

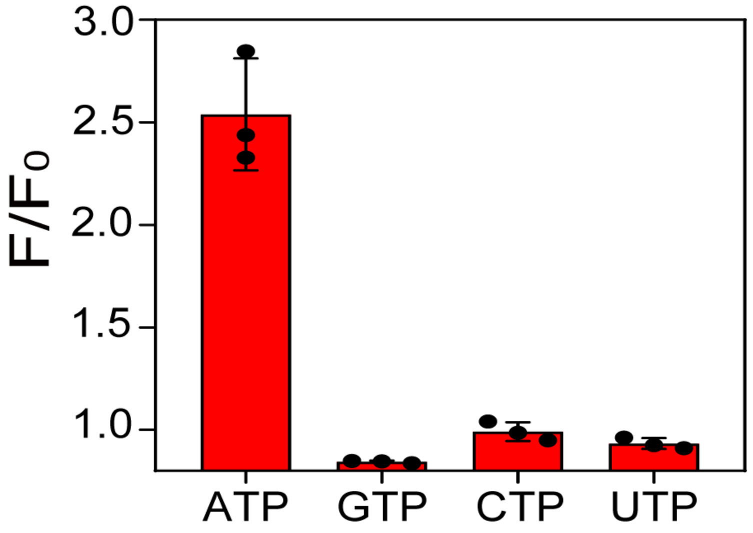

Supplementary Figure 23 | Selectivity studies of Cage-apt. The concentration of ATP and its analogues was 1 mM; Cage-apt-in-2 is 10 nM; Data are presented as mean values ± s.d. (n = 3).

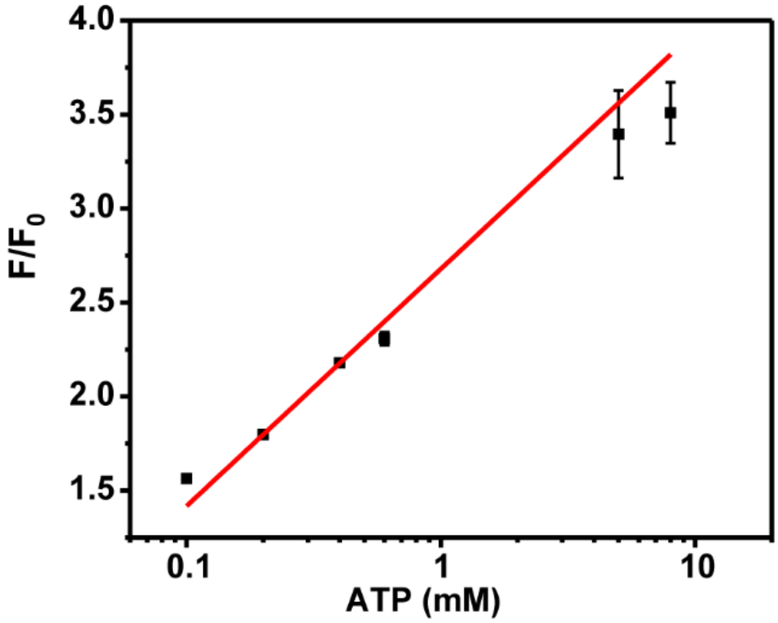

Supplementary Figure 24 | Standard curve for detection of ATP. Standard curve for detection of ATP from 0.1 mM to 10 mM; Data are presented as mean values ± s.d. (n = 3) (the value of x-axis is demonstrated in common logarithm mode based on the original data in Figure 5d).

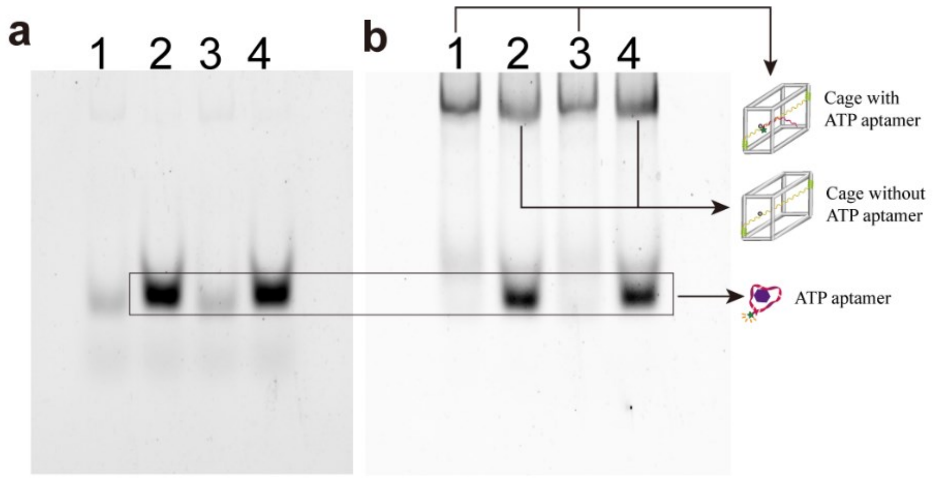

Supplementary Figure 25 | Responsive anlysis of Cage-apt-in-2 to ATP. Responsive analysis of Cage-apt-in-2 to ATP by 5% native PAGE: line 1, Cage-apt-in-2 (500 nM); line 2, Cage-apt-in-2 responsive to ATP (1mM); line 3, Cage-apt-in-2 (500nM) treated with 1 U mL^{-1} DNase I for 6h; line 4, Cage-apt-in-2 (500nM) responsive to ATP (1 mM) after treated with 1 U mL^{-1} DNase I. **a** The fluorescence (FAM) imaging of native-PAGE before staining with gel-green. **b** The fluorescence imaging of native-PAGE after staining with gel green. As the results shown, the FAM-labeled aptamers were apart from the DABCLY-labeled partially complementary strand (cDNA) in the presence of ATP, thus generating brighter band under the band of Cage (lane 2 and lane 4). Besides, the Cage-apt-in-2 still kept the same responsive ability with (lane 3, 4) and without (lane 1, 2) DNase I treated.

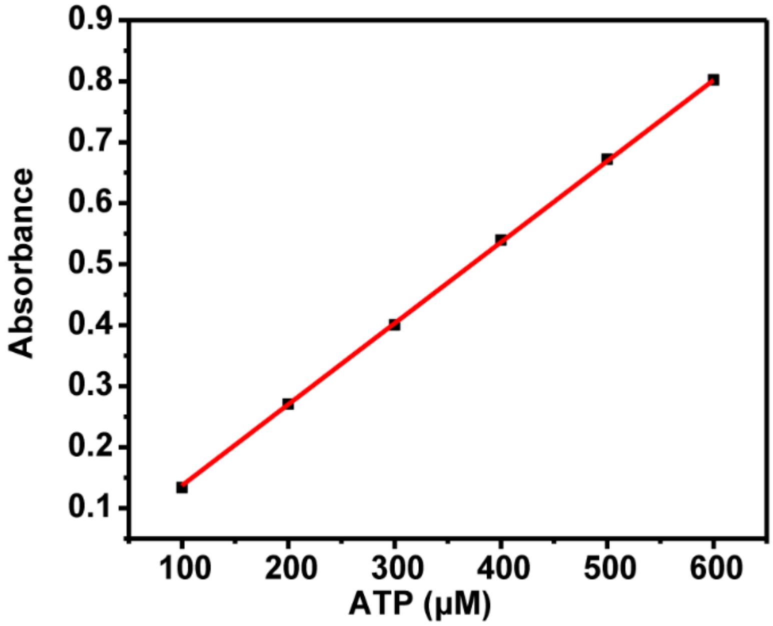

Supplementary Figure 26 | Standard curve for detection of ATP. Standard curve for detection of ATP by UV-vis absorption spectrometry. Data are presented as mean values ± s.d. (n = 3).

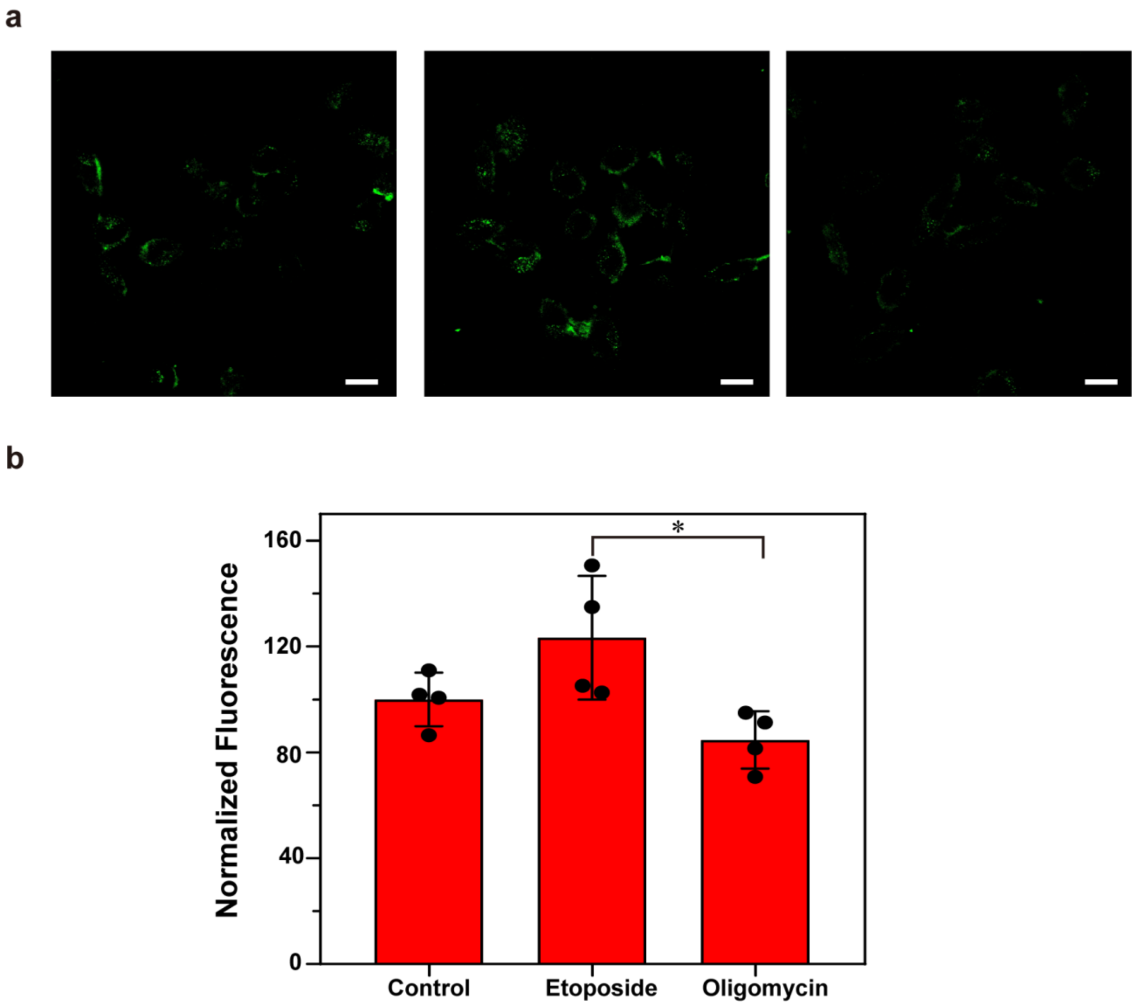

Supplementary Figure 27 | Fluorescent confocal imaging of endogenous ATP. a-b Confocal microscopy fluorescence images (**a**) and quantification result (**b**) for Cage-apt HeLa cells incubated with different drug treatment. From left to right: untreated cells, cells treated with 100 μM etoposide (a common activator of intracellular ATP), cells treated with 3 μg mL^{-1} oligomycin (a widely-used inhibitor for intracellular ATP)[1, 2]; Data are presented as mean values ± s.d. (n = 4); *p = 0.024< 0.05, by two-tailed unpaired Student's t-test. The scale bars are 20 μm.

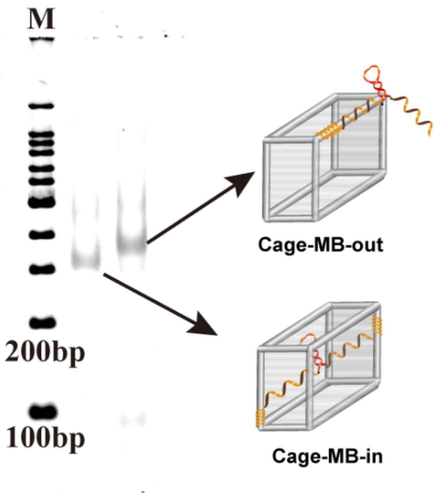

Supplementary Figure 28 | Native-PAGE analysis of Cage-MB-in and Cage-MB-out. Analysis of Cage-MB-in and Cage-MB-out assembly by 5% native-PAGE. Cage scaffold strands were the same as those of Cage 2.

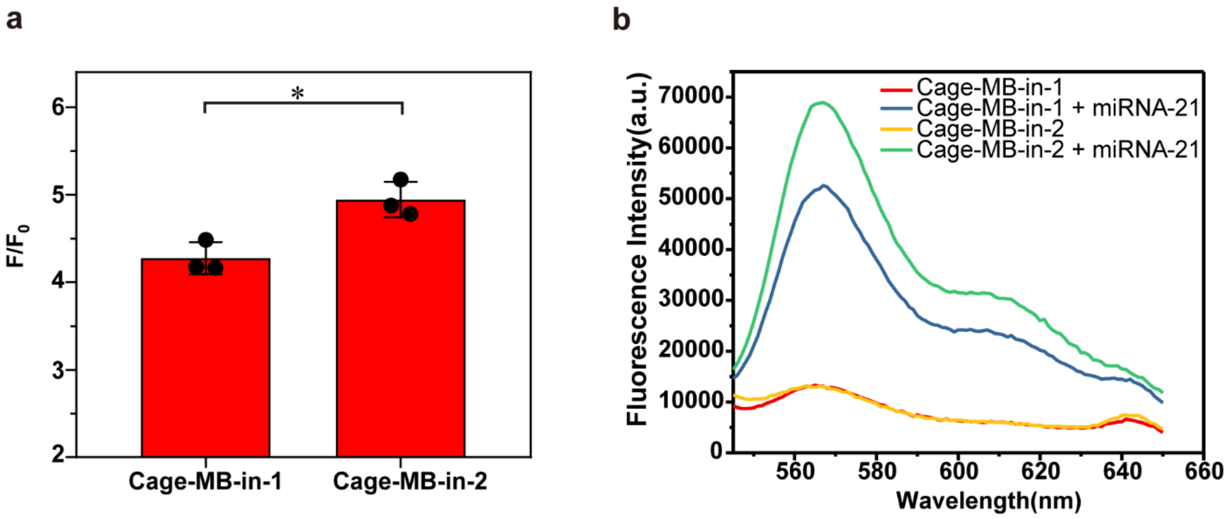

Supplementary Figure 29 | Comparison of biosensing ability between Cage-MB-in-1 and Cage-MB-in-2. a The fluorescence ratio of Cage-MB-in-1 and Cage-MB-in-2 (20 nM) with (F) and without (F_0) target micro RNA-21 (20 nM), Data are presented as mean values ± s.d. (n=3); *p = 0.013< 0.05, by two-tailed unpaired Student's t-test. **b** The fluorescence spectra corresponding to **a**.

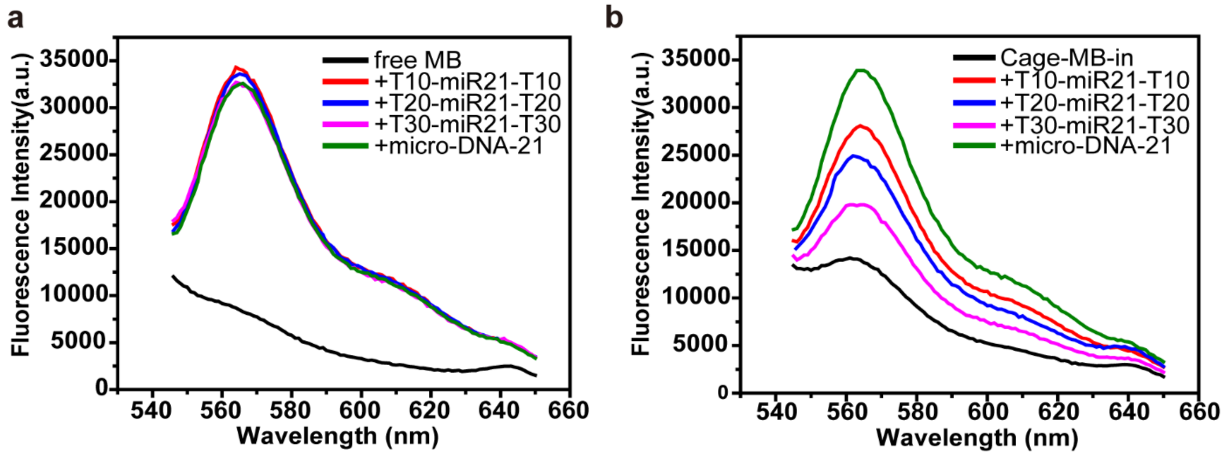

Supplementary Figure 30 | Size-selectivity ability of Cage-MB-in-2. Fluorescence response spectra of free MB (10 nM) (**a**) and Cage-MB-in-2 (10 nM) (**b**) to the nucleic acid targets with different lengths (10 nM).

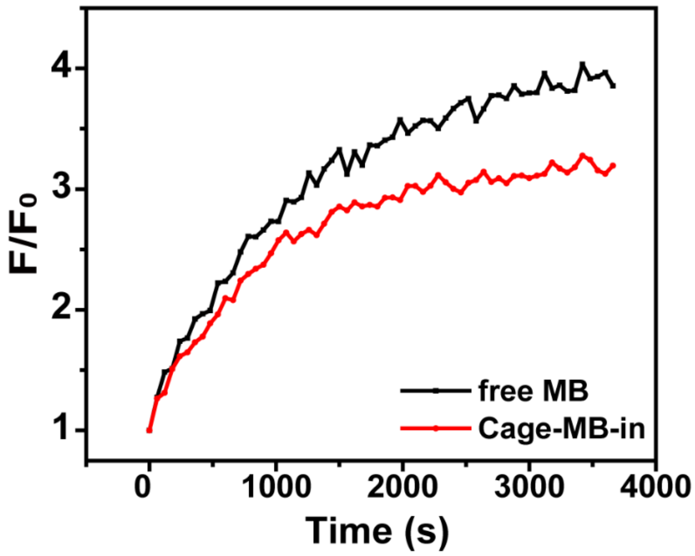

Supplementary Figure 31 | Response kinetics of Cage-MB-in. Comparison of response kinetics between free MB and Cage-MB-in (10 nM) in the presence of target.

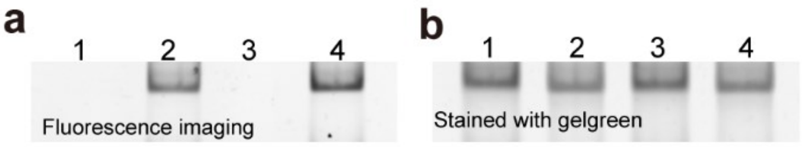

Supplementary Figure 32 | Responsive analysis of Cage-MB-in to microRNA-21-FAM by 5% native PAGE. Line 1, Cage-MB-in (500 nM); line 2, Cage-MB-in responsive to microRNA-21-FAM (500nM); line 3, Cage-MB-in (500 nM) treated with 1U mL^{-1} DNase I for 6h; line 4, Cage-MB-in (500 nM) responsive to microRNA-21-FAM (500 nM) after treated with 1U mL^{-1} DNase I. **a** The fluorescence (FAM) imaging of native-PAGE before staining with gel-green. **b** The fluorescence imaging of native-PAGE after staining with gel green. As the results shown, the band of Cage-MB-in brightened after recognizing the FAM-labeled *miRNA-21* (lane 2, 4), which means the *miRNA-21* can be recognized by the probe encapsulated in the DNA nanocages. Besides, the Cage-MB-in still kept the same responsive ability with (lane 3, 4) and without (lane 1, 2) DNase I treated.

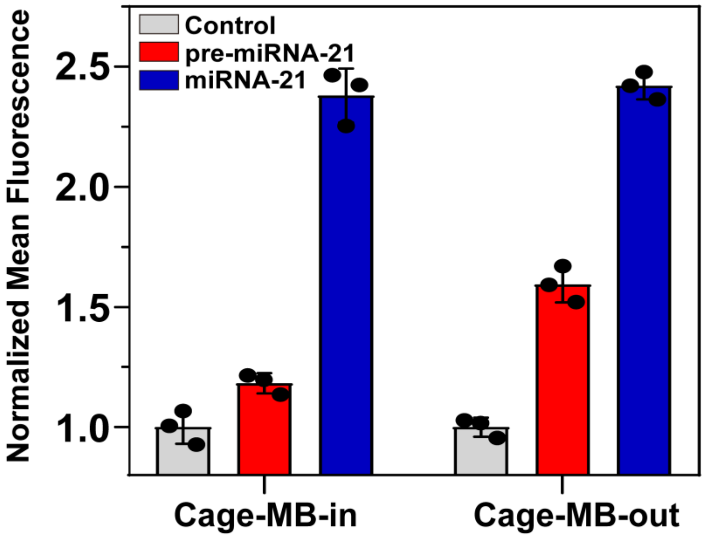

Supplementary Figure 33 | Size-selectivity biosensing of miRNA-21 by Cage-MB-in-2. Normalized mean fluorescence response of Cage-MB-in-2 (10 nM) and Cage-MB-out (10 nM) for *pre-miRNA-21* and mature *miRNA-21*. Data are presented as mean values ± s.d. (n = 3).

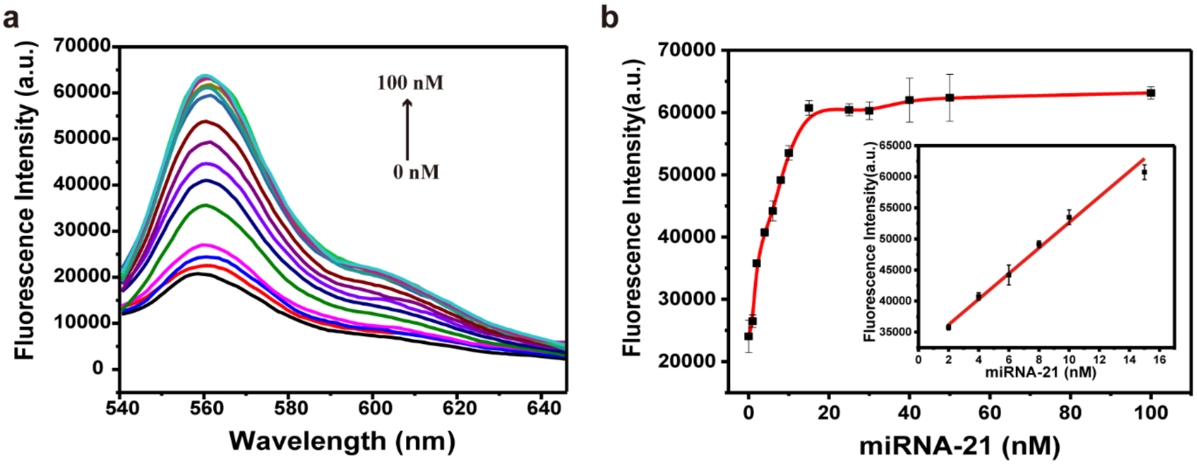

Supplementary Figure 34 | Fluorescence response of Cage-MB-in. a Fluorescence response of Cage-MB-in (20 nM) in the presence of different concentrations of mature *miRNA-21*, ranging from 0 to 100 nM. **b** Plot of the fluorescence intensity of Cy3 versus the target *miRNA-21* concentration. Inset: Linear correlation of the fluorescence intensity against concentrations of the *miRNA-21* target. The detection limit was estimated to be 0.62 nM (in terms of the rule of 3 times the standard deviation divided by the blank response).

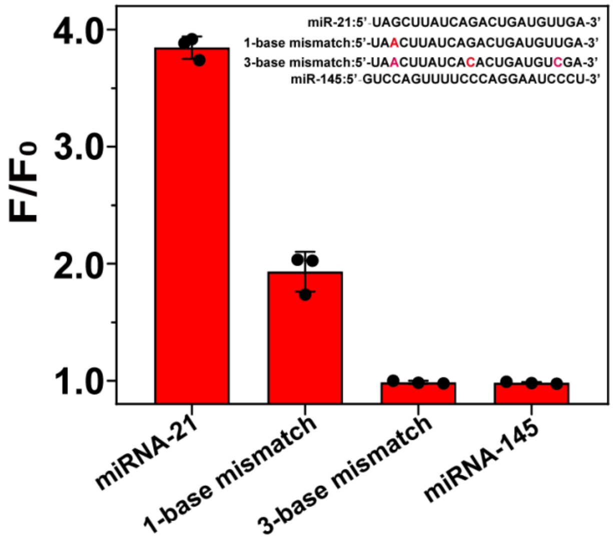

Supplementary Figure 35 | Selective experiment of Cage-MB-in. Selectivity studies of Cage-MB-in. The concentration of microRNA and other RNA sequences is 100 nM. F_0 and F refer to fluorescence intensity of probes in the absence and presence of targets, respectively. Data are presented as mean values ± s.d. (n = 3).

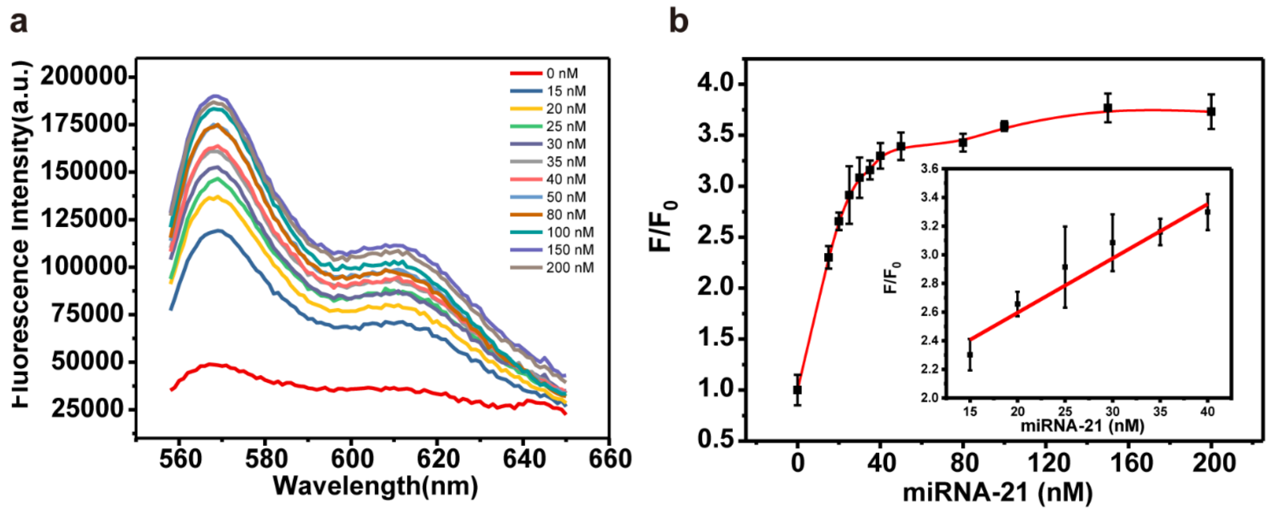

Supplementary Figure 36 | Fluorescence response of Cage-MB-in in human serum. **a** Fluorescence response of Cage-MB-in (20 nM) in the presence of different concentrations of mature *miRNA-21* in human serum, ranging from 0 to 200 nM **b** Plot of the fluorescence intensity of Cy3 versus the target *miRNA-21* concentration. Inset: Linear correlation of the fluorescence intensity against concentrations of the *miRNA-21* target. The detection limit was estimated to be 5.74 nM (in terms of the rule of 3 times the standard deviation divided by the blank response).

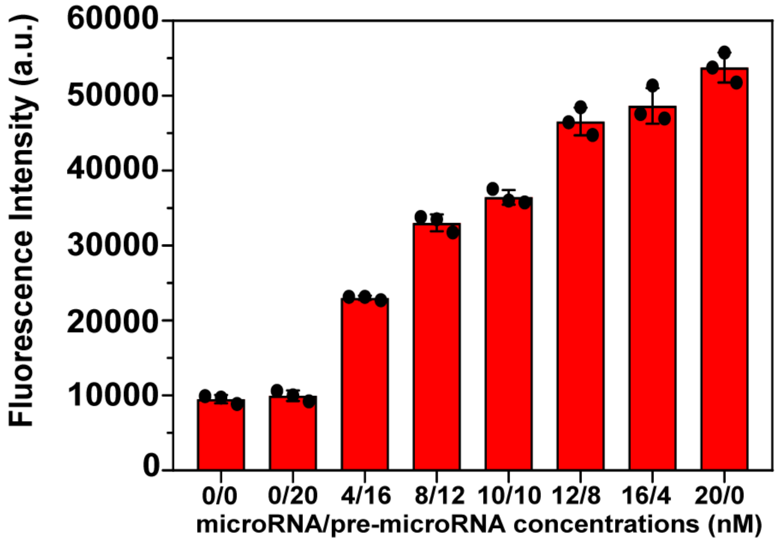

Supplementary Figure 37 | Fluorescence response of Cage-MB-in in mix samples. The Cage-MB-in (20 nM) were incubated with mixtures of mature and pre-miRNA targets at the indicated concentrations, where varied from 0-20 nM. Data are presented as mean values ± s.d. (n = 3).

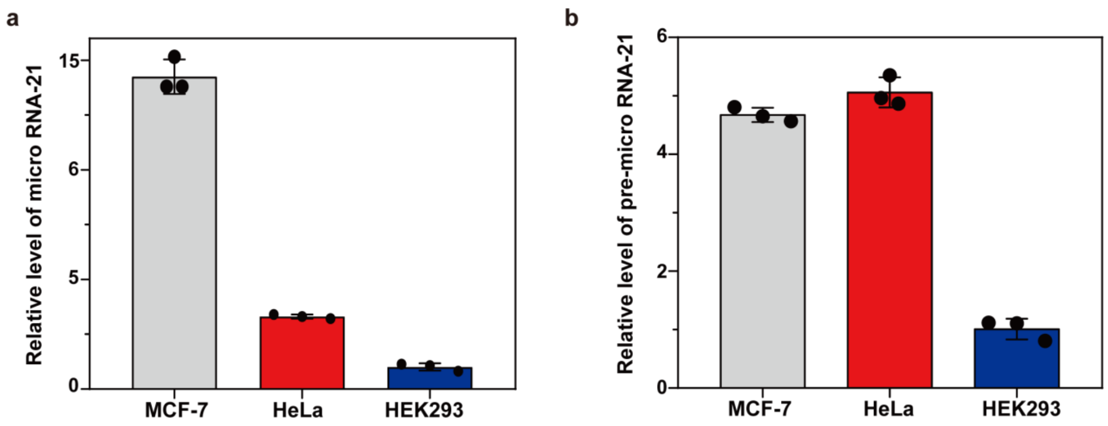

Supplementary Fig. 38 | qRT-PCR analysis of *miRNA-21* and *pre-miRNA-21* expression. a-b Relative expression levels of *miRNA-21* (**a**) and pre-*miRNA-21*(**b**) in HEK293, HeLa and MCF-7 cell lines, as estimated by qRT-PCR. Data are presented as mean values ± s.d. (n = 3).

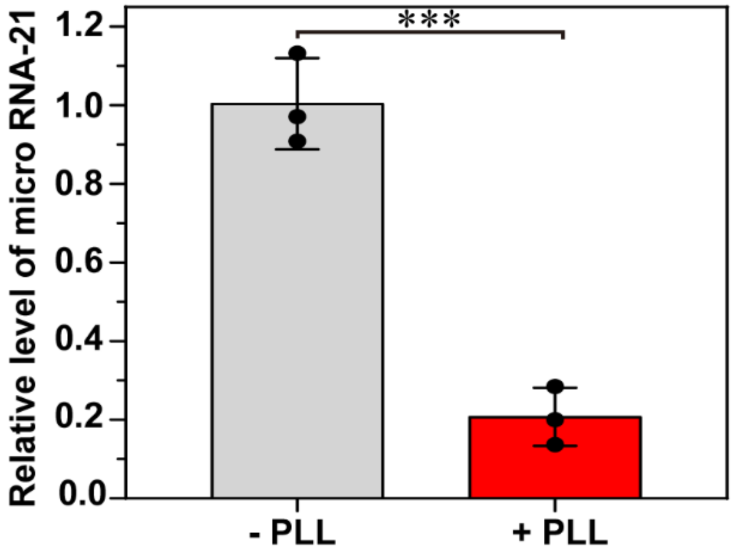

Supplementary Fig. 39 | qRT-PCR analysis of cells treated with PLL. Relative expression levels of *miRNA-21* before (-PLL) and after (+PLL) treatment with inhibitor poly-L-lysine. Data are presented as mean values ± s.d. (n=3); $***p = 0.00056 < 0.001$, by two-tailed unpaired Student's *t*-test.

Supplementary Tables

Supplementary Table 1 | ATP concentration was validated using Cage-apt-in-2 and UV-vis spectrum method.

Sample	ATP concentration (μM)	This method (μM)	UV-vis spectrum (μM)	*P*-value
1	100.00	100.93	101.11	0.93 (not significant)
2	150.00	154.45	154.74	0.92 (not significant)

Note: The data are provided as mean values (n=3). The *P*-value are calculated through comparing our method with UV-vis spectrum, by two-tailed unpaired Student's *t*-test.

Supplementary Table 2 | *MicroRNA-21* concentration (standard 5 nM) was validated using Cage-MB-in-2 (based on the standard curve in Supplementary Figure 34) and standard qRT-PCR method.

This method (nM)	qRT-PCR (nM)	*P*-value
4.22	4.26	0.97 (not significant)

Note: The data are provided as mean vlues (n=3). The *P*-value are calculated through comparing our method with UV-vis spectrum, by two-tailed unpaired Student's *t*-test.

Supplementary Table 3 | Sequences of oligonucleotides used in this work.

**Scaffold strands of Cage 1-Cage 5**		
Cage 1	C2-1	CCAGCCGCCGTTCCTGGATCCAAGGCTCTAGGTGTATTC AGGTAAGTGGCCATCCAAGCTGCGATCCGAC
	C1-2	CCACTCTGCTTTCTGGGATGCCATGACACAGTGATATTAC CTGAAT
	C1-3	GCCCCAGCATTGATGGTCTGCTTGTCGGATCGCAGCTTG GATGGTTTCACTGTGTC
	C2-4	TCTTCAGAGACAGCCAGGAGAATAAACAGAGGCCATGC TGGGGCCGTACAGTTCCAAAGGCATCCCAG
	C1-5	GCCTCTGTTTTTCCGTATATTCTTCGGCGGCTGGTTGCAG ACCATC
	C1-6	GAATATACGGTATCTCCTGGCTGTCTCTGAAGATTAGCAG AGTGGTTACCTAGAGCC
Cage 2	C2-1	CCAGCCGCCGTTCCTGGATCCAAGGCTCTAGGTGTATTC AGGTAAGTGGCCATCCAAGCTGCGATCCGAC
	C2-2	CCACTCCCGTTTCTGGGATGCCATACTCTAACTCAGATTC GCTGATATTACCTGAATTTTAGCGTTGGCT
	C2-3	GCCCCAGCATTGATAAGGATTTAGGTCAGCCCTTGTCGG ATCGCAGCTTGGATGGTTTCAGCGAATCTGAGTTAGAGT
	C2-4	TCTTCAGAGACAGCCAGGAGAATAAACAGAGGCCATGC TGGGGCCGTACAGTTCCAAAGGCATCCCAG

	C2-5	AATCCTTATCTTGCCTCTGTTTTTCCGTATATTCACGAAAA GGAGTTCGGCGGCTGGTTGGGCAGACCTA
	C2-6	CTCCTTTTCGTGAATATACGGTATCTCCTGGCTGTCTCTG AAGATTACGGGAGTGGAGCCAACGCTATTACCTAGAGCC
Cage 3	C3-1	GCTTGCCGTGGTGTCGGTCTGTTCCTGGATCCAAGGCTC TAGGTGTATTCAGGTAATGGACCCATAGGTGGCCATCCA AGCTGCGATCCGAC
	C3-2	CCACTCCCGTTTGTCCTCGCTCTCGTTGTCCTGATACTCT AACTCAGATTCGCTGATACTATGGGTCCATTACCTGAATT TTAGCGTTGGCT
	C2-3	GCCCCAGCATTGATAAGGATTTAGGTCAGCCCTTGTCGG ATCGCAGCTTGGATGGTTTCAGCGAATCTGAGTTAGAGT
	C3-4	TCTTCAGAGACAGCCAGGAGAATATAGACTAGGCATCAC AGTACCATGCTGGGGCCGTACAGTTCCAAACAGGACAA CGAGAGCGAGGAC
	C3-5	AATCCTTATCTTGTACTGTGATGCCTAGTCTATTTCCGTAT ATTCACGAAAAGGAGTTCAGACCGACACCACGGCAAGC TTGGGCAGACCTA
	C2-6	CTCCTTTTCGTGAATATACGGTATCTCCTGGCTGTCTCTG AAGATTACGGGAGTGGAGCCAACGCTATTACCTAGAGCC

Cage 4	C4-1-1	CGTGGTGTCGGTCTGTTCCTGGATCCAAGGCTCTAGGT GTATTCAGGTAATGGACC
	C4-1-2	CATAGGACGATGTCTGTGGCCATCCAAGCTGCGATCCG ACTACGAAGCCAGTGCTTGC
	C4-2-1	CCACTCCCGTTTTCGCCTCGCAGTCCTCGCTCTCGTTG TCCTGATACTCTAACTCA
	C4-2-2	GATTCGCTGATAAGGTATCGTCCTATGGGTCCATTACCT GAATTTTAGCGTTGCCT
	C2-3	GCCCCAGCATTGATAAGGATTTAGGTCAGCCCTTGTCG GATCGCAGCTTGGATGGTTTCAGCGAATCTGAGTTAGA GT
	C4-4-1	GAGGACTGCGAGGCGATTTCTTCAGAGACAGCCAGGA GAATATAGACTAGGCATCACA
	C4-4-2	GTACAGCTGCACCACATGCTGGGGCCGTACAGTTCCA AACAGGACAACGAGAGC
	C4-5-1	AATCCTTATCTTTGGTGCAGCTGTACTGTGATGCCTAGT CTATTTCCGTATATTC
	C4-5-2	ACGTCAAGGAGTTCAGACCGACACCACGGCAAGCAC TGGCTTCGTTGGGCTGACCTA
	C2-6	CTCCTTTTCGTGAATATACGGTATCTCCTGGCTGTCTCT GAAGATTACGGGAGTGGAGCCAACGCTATTACCTAGA GCC GCC

Cage 5	C5-1-1	CGTCGTGTCTGCCTGTTCCTGGATCCAAGGCTCTAGGT<br>GTCAGCGATCGTATTCAGGTAATGGACC
	C5-1-2	CATAGGACGATACGTGTGGCCATCCAAGCTGCGATCCG<br>ACGCAACTCTCCCGAATCCAGTGCTTAC
	C5-2-1	CACGTCCACTCCCGTTTTAGTGTCGCAGTCCTCGCTCT<br>GGATGTCCTGATACTCTATCTCTGATT
	C5-2-2	CGCTGACCTACTTCTGTAACGTATCGTCCTATGGGTCC<br>ATTACCTGAATTTTAGCGTTGGCTCGGTG
	C5-3-1	GCCCCAGCAACGCGATCACTTGATAAGGATTAGAGTA<br>GGTCTGCCGAGCGCTTGGAGAGTTGCGTCGGA
	C5-3-2	TCGCAGCTTGGATGGTTCAGAAGTAGGTCAGCGAATC<br>AGAGATAGAGT
	C5-4-1	GAGGACTGCGACACTATTCGAGTCAACATCTTCAGAG<br>ACAGCCAGGAGAATATAGACTAGGCATCA
	C5-4-2	CAGTACAGCTGCTCCACAGTGATCGCGTTGCTGGGGC<br>CGTACAGTTCCAAACAGGACATCCAGAGC
	C5-5-1	CTCTAATCCTTATCTTTGGTGCAGCTGTACTGTGATGCC<br>TAGTCTATTTCCGTATATTCACGAA

	C5-5-2	AAGGAGGAAGCTCACTTTCAGGCAGACACGACGGTA AGCACTGGCTTCGTTCGGCTCGGCAGACCTA
	C5-6-1	AGTGAGCTTCCTCCTTTTCGTGAATATACGGTATCTCC TGGCTGTCTCTG
	C5-6-2	AAGATGTTGACTCGTTACGGGAGTGGACGTGCACCG AGCCAACGCTATTGATCGCTGACACCTAGAGCC
**Scaffold strands of Czyme-in-2**		
C2-1		CCAGCCGCCGTTCCTGGATCCAAGGCTCTAGGTGTAT TCAGGTAAGTGGCCATCCAAGCTGCGATCCGAC
C2-2		CCACTCCCGTTTCTGGGATGCCATACTCTAACTCAGA TTCGCTGATATTACCTGAATTTTAGCGTTGGCT
C2-3		GCCCCAGCATTGATAAGGATTTAGGTCAGCCCTTGTC GGATCGCAGCTTGGATGGTTTCAGCGAATCTGAGTTA GAGT
C2-4		TCTTCAGAGACAGCCAGGAGAATAAACAGAGGCCAT GCTGGGGCCGTACAGTTCCAAAGGCATCCCAG
C2-5		AATCCTTATCTTGCCTCTGTTTTTCCGTATATTCACGA AAAGGAGTTCGGCGGCTGGTTGGGCAGACCTA
C2-6		CTCCTTTTCGTGAATATACGGTATCTCCTGGCTGTCTC TGAAGATTACGGGAGTGGAGCCAACGCTATTACCTAG AGCC
C-d-FAM-DABCLY		FAM-GAGGACAC(**rA**)GGAAGAGATG-DABCLY

C-DNAzyme-DABCLY	TGGAACTGTACGTACCCATCTCTTAACGGGGCTGTGCGGCTAGGAAGTAGTGTCCTC/iDabcyldT/ATCCATTGGATCCAGG
**Scaffold strands of Czyme-out-2**	
C2-1	CCAGCCGCCGTTCCTGGATCCAAGGCTCTAGGTGTATTCAGGTAAGTGGCCATCCAAGCTGCGATCCGAC
C2-2	CCACTCCCGTTTCTGGGATGCCATACTCTAACTCAGATTCGCTGATATTACCTGAATTTTAGCGTTGGCT
C2-3	GCCCCAGCATTGATAAGGATTTAGGTCAGCCCTTGTCGGATCGCAGCTTGGATGGTTTCAGCGAATCTGAGTTAGAGT
C2-4	TCTTCAGAGACAGCCAGGAGAATAAACAGAGGCCATGCTGGGGCCGTACAGTTCCAAAGGCATCCCAG
C2-5-out-1	AATCCTTATCTTGCCTCTGTTTTTCCGTATATTCA
C2-5-out-DNAzyme-DABCLY	CGAAAAGGAGTTCGGCGGCTGGTTGGGCAGACCTATTTTTCATCTCTTAACGGGGCTGTGCGGCTAGGAAGTAGTGTCCTC- DABCLY
C2-6	CTCCTTTTCGTGAATATACGGTATCTCCTGGCTGTCTCTGAAGATTACGGGAGTGGAGCCAACGCTATTACCTAGA
C-7-2	TGGAACTGTACGTACCGAATTCAGTTCAGAATTCATCCATTGGATCCAGG
**Free DNAzyme**	

L-histidine DNAzyme	CATCTCTTAACGGGGCTGTGCGGCTAGGAAGTAGTGTCCTC
**Scaffold strands of tetrahedron (TH-cDNA)**	
TH1	ACATTCCTAAGTCTGAAACATTACAGCTTGCTACACGAGAAGAGCCGCCATAGTA
TH2	TATCACCAGGCAGTTGACAGTGTAGCAAGCTGTAATAGATGCGAGGGTCCAATAC
TH3	TCAACTGCCTGGTGATAAAACGACACTACGTGGGAATCTACTATGGCGGCTCTTC
TH4-cDNA	TTCAGACTTAGGAATGTGCTTCCCACGTAGTGTCGTATGTATTGGACCCTCGCATTTTTTGAGGACACTACT
**Scaffold strands of Cage-apt**	
C2-1	CCAGCCGCCGTTCCTGGATCCAAGGCTCTAGGTGTATTCAGGTAAGTGGCCATCCAAGCTGCGATCCGAC
C2-2	CCACTCCCGTTTCTGGGATGCCATACTCTAACTCAGATTCGCTGATATTACCTGAATTTTAGCGTTGGCT
C2-3	GCCCCAGCATTGATAAGGATTTAGGTCAGCCCTTGTCGGATCGCAGCTTGGATGGTTTCAGCGAATCTGAGTTAGAGT
C2-4	TCTTCAGAGACAGCCAGGAGAATAAACAGAGGCCATGCTGGGGCCGTACAGTTCCAAAGGCATCCCAG
C2-5	AATCCTTATCTTGCCTCTGTTTTTCCGTATATTCACGAAAAGGAGTTCGGCGGCTGGTTGGGCAGACCTA

C2-6	CTCCTTTTCGTGAATATACGGTATCTCCTGGCTGTCTCTGAAGATTACGGGAGTGGAGCCAACGCTATTACCTAGAGCC
C-cDNA-DABCLY	TGGAACTGTACGTACCACTCCCCCAGGT/iDabcyldT/TAGCTATCCATTGGATCCAGG
C-ATP aptamer	FAM-ACCTGGGGGAGTATTGCGGAGGAAGGT
**Scaffold strands of Cage-MB-in**	
C2-1	CCAGCCGCCGTTCCTGGATCCAAGGCTCTAGGTGTATTCAGGTAAGTGGCCATCCAAGCTGCGATCCGAC
C2-2	CCACTCCCGTTTCTGGGATGCCATACTCTAACTCAGATTCGCTGATATTACCTGAATTTTAGCGTTGGCT
C2-3	GCCCCAGCATTGATAAGGATTTAGGTCAGCCCTTGTCGGATCGCAGCTTGGATGGTTTCAGCGAATCTGAGTTAGAGT
C2-4	TCTTCAGAGACAGCCAGGAGAATAAACAGAGGCCATGCTGGGGCCGTACAGTTCCAAAGGCATCCCAG
C2-5	AATCCTTATCTTGCCTCTGTTTTTCCGTATATTCACGAAAAGGAGTTCGGCGGCTGGTTGGGCAGACCTA
C2-6	CTCCTTTTCGTGAATATACGGTATCTCCTGGCTGTCTCTGAAGATTACGGGAGTGGAGCCAACGCTATTACCTAGAGCC

C7-MB-21	TGGAACTGTACGTACCCATCTCTTCT/iCy3dT/GCTCGTCAACATCAGTCTGATAAGCTACGAGC/iBHQ2dT/GTGTACTCATTTATTGGATCCAGG
**Scaffold strands of Cage-MB-out**	
C2-1	CCAGCCGCCGTTCCTGGATCCAAGGCTCTAGGTGTATTCAGGTAAGTGGCCATCCAAGCTGCGATCCGAC
C2-2	CCACTCCCGTTTCTGGGATGCCATACTCTAACTCAGATTCGCTGATATTACCTGAATTTAGCGTTGGCT
C2-3	GCCCCAGCATTGATAAGGATTTAGGTCAGCCCTTGTCGGATCGCAGCTTGGATGGTTTCAGCGAATCTGAGTTAGAGT
C2-4	TCTTCAGAGACAGCCAGGAGAATAAACAGAGGCCATGCTGGGGCCGTACAGTTCCAAAGGCATCCCAG
C2-5-out-1	AATCCTTATCTTGCCTCTGTTTTTCCGTATATTCA
C2-5-out-cap	CGAAAAGGAGTTCGGCGGCTGGTTGGGCAGACCTATTTTTCCTGGATCCAATAAATGAGT
C2-6	CTCCTTTTCGTGAATATACGGTATCTCCTGGCTGTCTCTGAAGATTACGGGAGTGGAGCCAACGCTATTACCTAGAGCC
C7-MB-21	TGGAACTGTACGTACCCATCTCTTCT/iCy3dT/GCTCGTCAACATCAGTCTGATAAGCTACGAGC/iBHQ2dT/GTGTACTCATTTATTGGATCCAGG
***miRNA-21* mimicry of sequences**	

miRNA-21	TAGCTTATCAGACTGATGTTGA
miRNA-21-FAM	FAM-TAGCTTATCAGACTGATGTTGA
T10-miR21-T10	TTTTTTTTTTTAGCTTATCAGACTGATGTTGATTTTTTTTTT
T20-miR21-T20	TTTTTTTTTTTTTTTTTTTTTAGCTTATCAGACTGATGTTGATTTTTTTTTTTTTTTTTTTT
T30-miR21-T30	TTTTTTTTTTTTTTTTTTTTTTTTTTTTTTTAGCTTATCAGACTGATGTTGATTTTTTTTTTTTTTTTTTTTTTTTTTTTTT
**The target RNA sequence of *miRNA-21* and *pre-miRNA-21***	
Mature *miRNA-21*	UAGCUUAUCAGACUGAUGUUGA
Pre-*micro RNA-21*	UGUCGGAUAGCUUAUCAGACUGAUGUUGACUGUUGAAUCUCAUGGCAACACCAGUCGAUGGGCUUACUGACA
**The control microRNA**	
1-base-mismatch	UAACUUAUCAGACUGAUGUUGA
3-base-mismatch	UAACUUAUCACACUGAUGUCGA
miR-145	GUCCAGUUUUCCCAGGAAUCCCU
**Primer sequences**	
U6 snRNA-FO	CGCTTCGGCAGCACATATAC
U6 snRNA-RE	TTCACGAATTTGCGTGTCATC
Mature *micro RNA-21*-FO	TCGCCCGTAGCTTATCAGACT

Mature *micro RNA-21*-RE	CAGAGCAGGGTCCGAGGTA
Pre-microRNA-21-FO	GTCGGGTAGCTTATCAGACTGA
Pre-microRNA-21-RE	GTCAGACAGCCCATCGACT

Note: For Cage 4 and Cage 5, we split the long strand (over 90 nt) into two DNA strands for more efficient assembly. For DNAzyme and *miRNA-21* probe modified on the surface frame of DNA (Czyme-out-2 and Cage-MB-out, respectively), we split the C2-5 into two DNA strands (C2-5-out-1 and C2-5-out-DNAzyme-DABCYL for Czyme-out-2, C2-5-out-cap for capturing the *miRNA-21*) and designed the C-7-2 to maintain the rigidity of the cage. For sequence of "C-d-FAM-DABCLY", the blot font indicates the RNA site. For the sequences of "The control microRNA", the red font indicates the mismatched base sites.

Supplementary References

1. Izyumov, D.S. et al. "Wages of Fear": transient threefold decrease in intracellular ATP level imposes apoptosis. Biochim. Biophys. Acta. **1658**, 141-147 (2004).
2. Wu, C. et al. Engineering of switchable aptamer micelle flares for molecular imaging in living cells. ACS Nano **7**, 5724-5731 (2013).
