# Supplementary material for: Size-selective molecular recognition based on a confined DNA molecular sieve using cavity-tunable framework nucleic acids
Source: Nat Commun. 2020 Mar 23;11:1518. doi: 10.1038/s41467-020-15297-7 (PMC7089997; doi:10.1038/s41467-020-15297-7)
Supplement: Supplementary file 2 — Reporting Summary [file 41467_2020_15297_MOESM2_ESM.pdf]

nature research

Corresponding author(s): Xiaobing Zhang

Last updated by author(s): Feb 10, 2020

Reporting Summary

Nature Research wishes to improve the reproducibility of the work that we publish. This form provides structure for consistency and transparency in reporting. For further information on Nature Research policies, see Authors & Referees and the Editorial Policy Checklist .

Statistics

For all statistical analyses, confirm that the following items are present in the figure legend, table legend, main text, or Methods section.

n/a	Confirmed	
☐	☒	The exact sample size (*n*) for each experimental group/condition, given as a discrete number and unit of measurement
☐	☒	A statement on whether measurements were taken from distinct samples or whether the same sample was measured repeatedly
☐	☒	The statistical test(s) used AND whether they are one- or two-sided<br>*Only common tests should be described solely by name; describe more complex techniques in the Methods section.*
☒	☐	A description of all covariates tested
☒	☐	A description of any assumptions or corrections, such as tests of normality and adjustment for multiple comparisons
☐	☒	A full description of the statistical parameters including central tendency (e.g. means) or other basic estimates (e.g. regression coefficient) AND variation (e.g. standard deviation) or associated estimates of uncertainty (e.g. confidence intervals)
☐	☒	For null hypothesis testing, the test statistic (e.g. *F*, *t*, *r*) with confidence intervals, effect sizes, degrees of freedom and *P* value noted<br>*Give P values as exact values whenever suitable.*
☒	☐	For Bayesian analysis, information on the choice of priors and Markov chain Monte Carlo settings
☒	☐	For hierarchical and complex designs, identification of the appropriate level for tests and full reporting of outcomes
☒	☐	Estimates of effect sizes (e.g. Cohen's *d*, Pearson's *r*), indicating how they were calculated

Our web collection on statistics for biologists contains articles on many of the points above.

Software and code

Policy information about availability of computer code

Data collection	For quantitative analysis of oligonucleotides, Thermo Scientific NanoDrop 2000c Spectrophotometer was utilized. Fluorescence spectral data was measured on Fluoro Max-4 (Horiba Jobin Yvon). PAGE gel imaging data was collected using Bio-Rad ChemiDoc XRS System. Confocal imaging data was collected on Zeiss LSM 880 and Olympus FV1000 confocal laser scanning microscope. Absorption spectrometry data was collected on UV-2600 (Shimadzu, Japan). Dynamic light scattering (DLS) were measured on the Malvern Zetasizer Nano ZS90 (Malvern Instruments, Ltd., Worcestershire, UK). qRT-PCR data was collected on Applied Biosystem 7000.The AFM characterization of the sample was carried out on a Bruker Multimode V8 Scanning Probe Microscope
Data analysis	Fluorescence spectral data was analyzed with OriginLab 2017. Statistical mean and differences were evaluated using Microsoft excel 2013's statistical tools and GraphPad Prism 8.0.1. Confocal imaging data was analyzed using Image J.

For manuscripts utilizing custom algorithms or software that are central to the research but not yet described in published literature, software must be made available to editors/reviewers. We strongly encourage code deposition in a community repository (e.g. GitHub). See the Nature Research guidelines for submitting code & software for further information.

Data

Policy information about availability of data

All manuscripts must include a data availability statement. This statement should provide the following information, where applicable:

- Accession codes, unique identifiers, or web links for publicly available datasets
- A list of figures that have associated raw data
- A description of any restrictions on data availability

The authors declare that the main data supporting the findings of this study are available within the article and its Supplementary Information files. Extra data are available from the corresponding author upon request.

Field-specific reporting

Please select the one below that is the best fit for your research. If you are not sure, read the appropriate sections before making your selection.

[x] Life sciences [ ] Behavioural & social sciences [ ] Ecological, evolutionary & environmental sciences

For a reference copy of the document with all sections, see nature.com/documents/nr-reporting-summary-flat.pdf

Life sciences study design

All studies must disclose on these points even when the disclosure is negative.

Sample size	All analyses involve at least 3 independent samples. Such sample size was chosen with referral to similar studies previously
Data exclusions	No data was excluded from studies.
Replication	All analyses were repeated at least three times with n>=3; All replication of experiments was successful, however, in the case of replication studies of nuclease (DNase I) digestion and protein absorption experiment (Fig. 4e & Fig. 4f), replication need more carefully due to the activity fluctuations of enzyme and protein. So when we conduct the experiments, we determined the alteration of fluorescence of each sample at the same time to reduce interference from enzyme or protein activity.
Randomization	Throughout the whole experiment, samples were randomized into groups.
Blinding	No blinding was used throughout experiments. All data collected was quantifiable and blinding would not change any bias in data collected.

Reporting for specific materials, systems and methods

We require information from authors about some types of materials, experimental systems and methods used in many studies. Here, indicate whether each material, system or method listed is relevant to your study. If you are not sure if a list item applies to your research, read the appropriate section before selecting a response.

Materials & experimental systems

n/a	Involved in the study	
[x]	[ ]	Antibodies
[ ]	[x]	Eukaryotic cell lines
[x]	[ ]	Palaeontology
[x]	[ ]	Animals and other organisms
[x]	[ ]	Human research participants
[x]	[ ]	Clinical data

Methods

n/a	Involved in the study	
[x]	[ ]	ChIP-seq
[x]	[ ]	Flow cytometry
[x]	[ ]	MRI-based neuroimaging

Eukaryotic cell lines

Policy information about cell lines

Cell line source(s)	HeLa ,MCF-7 and HEK293 cells were obtained from ATCC.
Authentication	Cell cultures purchased from ATCC were authenticated by Short Tandem Repeat (STR) prior to purchase
Mycoplasma contamination	Cell lines were not tested for mycoplasma contamination
Commonly misidentified lines (See ICLAC register)	No misidentified line for HeLa, MCF-7 and HEK293
